# Nitrate Enhances Gastric Mucosa Defense and Repair Process in Ethanol‐Induced Gastric Ulcer Rats via the Notch–Tff2 Pathway

**DOI:** 10.1002/mco2.70628

**Published:** 2026-02-05

**Authors:** Ying Liu, Xin Wen, Yuxuan Lin, Chunmei Zhang, Jinsong Wang, Guangyong Sun, Dong Zhang, Renhong Yan, Mo Chen, Songlin Wang, Shaorong Li

**Affiliations:** ^1^ Salivary Gland Disease Center and Beijing Key Laboratory of Tooth Regeneration and Function Reconstruction, Beijing Laboratory of Oral Health and School of Stomatology, Capital Medical University Beijing China; ^2^ Immunology Research Center For Oral and Systemic Health Beijing Friendship Hospital, Capital Medical University Beijing China; ^3^ Department of Biochemistry and Molecular Biology School of Basic Medical Sciences, Capital Medical University Beijing China; ^4^ Medical Research Center Beijing Institute of Respiratory Medicine and Beijing Chao‐Yang Hospital, Capital Medical University Beijing China; ^5^ Department of Biochemistry School of Medicine, Southern University of Science and Technology Shenzhen Guangdong Province China; ^6^ Key University Laboratory of Metabolism and Health of Guangdong, SUSTech Homeostatic Medicine Institute, Institute for Biological Electron Microscopy, Southern University of Science and Technology Shenzhen Guangdong Province China; ^7^ Department of Pharmacology Joint Laboratory of Guangdong‐Hong Kong Universities for Vascular Homeostasis and Diseases, School of Medicine and SUSTech Homeostatic Medicine Institute, Southern University of Science and Technology Shenzhen China; ^8^ Laboratory For Oral and General Health Integration and Translation Beijing Tiantan Hospital, Capital Medical University Beijing China; ^9^ Laboratory of Homeostatic Medicine School of Medicine, Southern University of Science and Technology Shenzhen China; ^10^ Department of Endodontics School of Stomatology Capital Medical University Beijing China

**Keywords:** epithelial restitution, ethanol, gastric mucosal barrier, migration, Notch, trefoil factor 2

## Abstract

Gastric mucosal integrity is essential for maintaining systemic homeostasis, serving as the primary defense against external insults. Ethanol ingestion is a major clinical cause of gastric mucosal injury, yet effective prevention or treatment remains limited. This study investigates the protective role of nitrate against ethanol‐induced gastric ulcers and its underlying mechanisms. In vivo, nitrate significantly ameliorated ethanol‐induced gastric bleeding, edema, inflammation, and mucus layer thinning in rats, while strengthening the vascular endothelial barrier. Transcriptomic analyses and trefoil factor 2 (Tff2)‐knockdown rats experiment identified *Tff2* as the key gene responsible for mediating nitrate's protective effects against ethanol. In vitro, TFF2 was found to be a crucial target for nitrates, which enhance the migratory reparative capacities of human gastric epithelial cells. Further assays revealed that RBPJ regulates the TFF2 promoter, and NICD–RBPJ complex formation is critical for TFF2 transcriptional repression. We demonstrate for the first time that TFF2 is a central effector in nitrate‐mediated gastric mucosal defense and repair and implicate the Notch signaling pathway in TFF2 regulation. These findings suggest nitrate exerts a protective effect on the gastric mucosa through multiple ways. TFF2 modulation as a potential preventive strategy for ethanol‐induced gastric ulcers.

## Graphical abstract

Nitrate protects the three major barriers of the gastric mucosa, alleviating bleeding and inflammation of ethanol‐induced gastric ulcer in rats, and promotes migration of gastric epithelial cells, accelerating the restoration of the ulcerated epithelium, which is strongly related to TFF2, a gastric mucosal protective factor negatively regulated by the Notch signaling pathway.

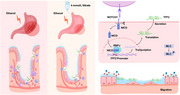

## 1 Introduction

Gastric ulcer (GU) is one of the most common diseases [1] with a notably high incidence [2] and a recurrent tendency [3, 4, 5]. Its etiology mainly includes nonsteroidal anti‐inflammatory drugs (NSAIDs), bacteria, alcohol (ethanol, EtOH), and stress [6, 7]. As the primary organ directly exposed to and absorbing EtOH [8, 9], the prevalence rate of EtOH‐induced ulcers remains notably high [10]. Ulcers are key factors in upper gastrointestinal bleeding [11] and can lead to gastric perforation [12]. However, research on the mechanisms, prevention, and treatment strategies of EtOH‐induced GUs remains limited. Conventional clinical treatments primarily include antibiotics and proton‐pump inhibitors [13] but lack targeted approaches. Given the substantial increase in EtOH consumption, more attention should be drawn to EtOH‐induced GUs.

Gastric mucosa integrity is crucial in gastrointestinal homeostasis, encompassing the pre‐epithelial, epithelial, and sub‐epithelial defense [14, 15]. EtOH directly impairs mucosal defense in a dose‐dependent manner. The mucus barrier builds the pre‐epithelial defense, and high EtOH concentrations can penetrate the mucus barrier [16]. The absence of mucus protection renders epithelial cells (epithelial defense) vulnerable to luminal irritants, such as gastric acid, pepsin, and bacteria [17, 18], which not only disrupts epithelium continuity but also triggers proinflammatory factors and vasoconstrictors release, further exacerbating mucosal damage [19]. Simultaneously, EtOH increases vascular permeability, disrupting the blood‐flow barrier (sub‐epithelial defense) [20, 21]. EtOH can prolong stomach exposure to irritants by slowing gastric emptying [22] and inducing pyloric spasms [23], leading to additional harm. Upon impairment of all three barriers, the gastric mucosa presents with ischemia, hemorrhage, oedema, mucus layer breakage, and inflammation.

To restore gastric barrier function, the initial step is to reconstitute epithelial continuity, primarily by initiating cell migration to damaged areas [24]. This early repair process, known as “restitution,” can be initiated within 3 min postinjury [25]. Generally, proliferation, differentiation and regeneration are too slow to effectively protect the mucosa in the early stages [26]. Swift repair is crucial for preventing continued exposure of damaged sites to luminal irritants and serves as a vital intervention before extensive inflammation [27]. Although restitution is a multistep process, cell migration is a critical step of early gastric mucosal repair.

Nitrate is an essential nutrient that is mainly derived from vegetables. Dietary nitrate is an important way of exogenous nitrate supplementation [28]. Emerging evidence highlights the pivotal role of dietary nitrate in maintaining organism homeostasis [29, 30]. Numerous studies have demonstrated that nitrate supplementation exhibits significant protective effects against various tissue injuries and multisystem disorders [31, 32, 33, 34, 35], particularly in safeguarding the digestive system. Notably, nitrate demonstrates remarkable efficacy in protecting both digestive glands [36, 37, 38] and the gastrointestinal tract [39, 40]. Among these protective effects, its beneficial impact on gastric health has been extensively investigated and well documented [41, 42]. It has been reported that dietary nitrate confers gastric protection against NSAIDs and stress‐induced GUs [43, 44, 45]. There is a lack of research regarding the effects of nitrate on EtOH‐induced GU.

This study investigated the protective efficacy of nitrate against EtOH‐induced GU and its role in mucosal repair, aiming to identify key targets and elucidate the mechanisms. With in vivo and in vitro models employed, the main findings were obtained as following: TFF2 is a key target for nitrate to resist EtOH and plays a dual role of strengthening the gastric mucosa barriers and promoting early repair of the gastric mucosal epithelium. Furthermore, the Notch signaling pathway is one of the key pathways by which nitrate regulates the expression of TFF2. Our work provides mechanical insights for future clinical strategies by revealing new nitrate targets and addresses a key gap in the upstream regulation of TFF2.

## 2 Results

### 2.1 Dietary Nitrate Attenuates EtOH‐Induced Gastric Mucosal Hemorrhage and Oedema In Vivo

**FIGURE 1:**
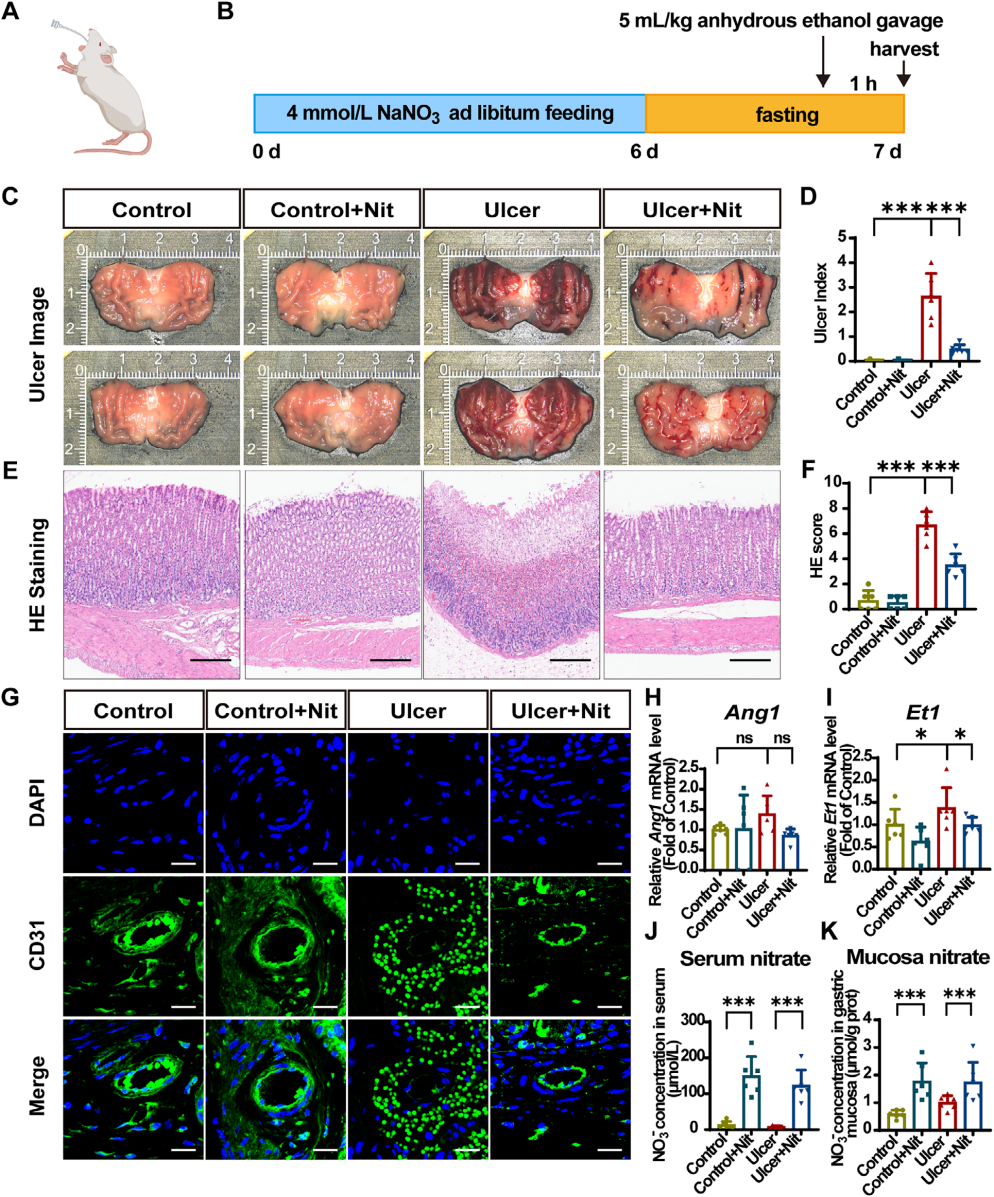
Dietary nitrate attenuates ethanol‐induced gastric mucosal hemorrhage and oedema in vivo. (A) Establishment of ethanol‐induced gastric ulcer in rats by intragastric administration of anhydrous ethanol. (B) Flowchart of the animal experimental procedures. (C) Macroscopic appearance of the gastric mucosa in four groups. (D) Quantification of gastric damage expressed as the ulcer index (UI). The UI was calculated as follows: UI = 10 × (ulcerated area/total mucosal area). (E) Representative HE‐stained images of stomach tissues. Scale bar = 50 µm. (F) Histological evaluation of gastric lesions using a microscopic HE score (0–14). The score sums the severity of four features: inflammatory cells (0–3), mucosal edema (0–4), hemorrhage (0–4), and epithelial loss (0–3). (G) Representative CD31 IF staining images in gastric mucosa. Scale bar = 20 µm. (H and I) RT‐qPCR analysis of *Ang1* and *Et1* mRNA expression in gastric mucosa. Target gene expression was normalized to *Gapdh* mRNA and expressed as fold change relative to the control group. (J and K) The nitrate levels of the serum and gastric mucosa in the four groups. Quantitative data are expressed as the mean ± SD. *p < 0.05, ***p < 0.001, and ns denotes no significance. HE, hematoxylin–eosin; Nit, nitrate; IF, immunofluorescence; CD31, platelet endothelial cell adhesion molecule‐1; Ang1, angiotensin‐1; Et1, endothelin‐1; RT‐qPCR, real‐time quantitative polymerase chain reaction; DAPI, 2‐(4‐amidinophenyl)‐6‐indolecarbamidine dihydrochloride; SD, standard deviation.

The rats were randomized into four groups: control, control + Nit (nitrate), ulcer, and ulcer + Nit. The nitrate‐supplemented groups were given 4 mmol/L nitrate water for 7 days in advance. GUs were induced by gavage administration of anhydrous EtOH (5 mL/kg body weight), and the rats were sacrificed 1 h after gavage for sample collection (Figure 1A,B).

Macroscopically, nitrate significantly alleviated EtOH‐induced gastric damage. The EtOH‐exposed rats showed broad, thick, and dull red stripes along the long axis with severe erosion and oedema of the gastric mucosa compared with the control rats. In the ulcer + Nit group, gastric tissue hemorrhage and oedema were noticeably reduced. The ulcer index was significantly reduced (Figure 1C,D). Hematoxylin–eosin (HE) staining was performed to ascertain the antiulcer effects of nitrate. The slices were evaluated based on gastric mucosa integrity, oedema, erythrocytes congestion, and neutrophil infiltration. The control group, with or without nitrate, displayed a normal gastric mucosal tissue structure, whereas the gastric mucosa in the ulcer group was disrupted, accompanied by significant infiltration of erythrocytes and inflammatory cells. The ulcer + Nit group showed a significant reduction in the aforementioned cells, alleviated oedema, and reduced structural loss (Figure 1E,F). In conclusion, preventive intake of 4 mmol/L nitrate water can reduce EtOH‐induced gastric mucosal damage.

EtOH increases vascular permeability, leading to microvascular damage and blood flow stasis. Immunofluorescence (IF) staining of the vascular endothelial marker CD31 in rat gastric tissue revealed that the control group had a continuous and intact vascular wall beneath the gastric mucosal epithelium, whereas the ulcer group exhibited a damaged vascular endothelium, with disrupted continuity, increased permeability, and the staining of CD31 in erythrocytes showed a granular distribution infiltrating the perivascular connective tissue. Nitrate supplementation markedly enhanced the vascular endothelial integrity, reducing extravasation of erythrocytes. (Figure 1G). To determine mucosal blood flow, we also assessed endothelin and angiotensin expression, with higher expression indicating lower blood flow. The ulcer group had elevated endothelin‐1 (*Et1*) and angiotensin‐1 (*Ang1*) mRNA level in comparison with the control group, whereas the ulcer + Nit group exhibited a decreasing tendency (Figure 1H,I). In summary, we conclude that nitrate promotes blood flow and exerts antiulcer effects by protecting the vascular endothelium and dilating vessels. To further confirm the action of nitrate, we measured nitrate levels in the serum and gastric mucosa and found that nitrate supplementation significantly increased the nitrate levels in both (Figure 1J,K).

### 2.2 Dietary Nitrate Stabilizes the Gastric Mucus Layer and Improves Mucosal Healing of EtOH‐Induced Injury In Vivo

**FIGURE 2:**
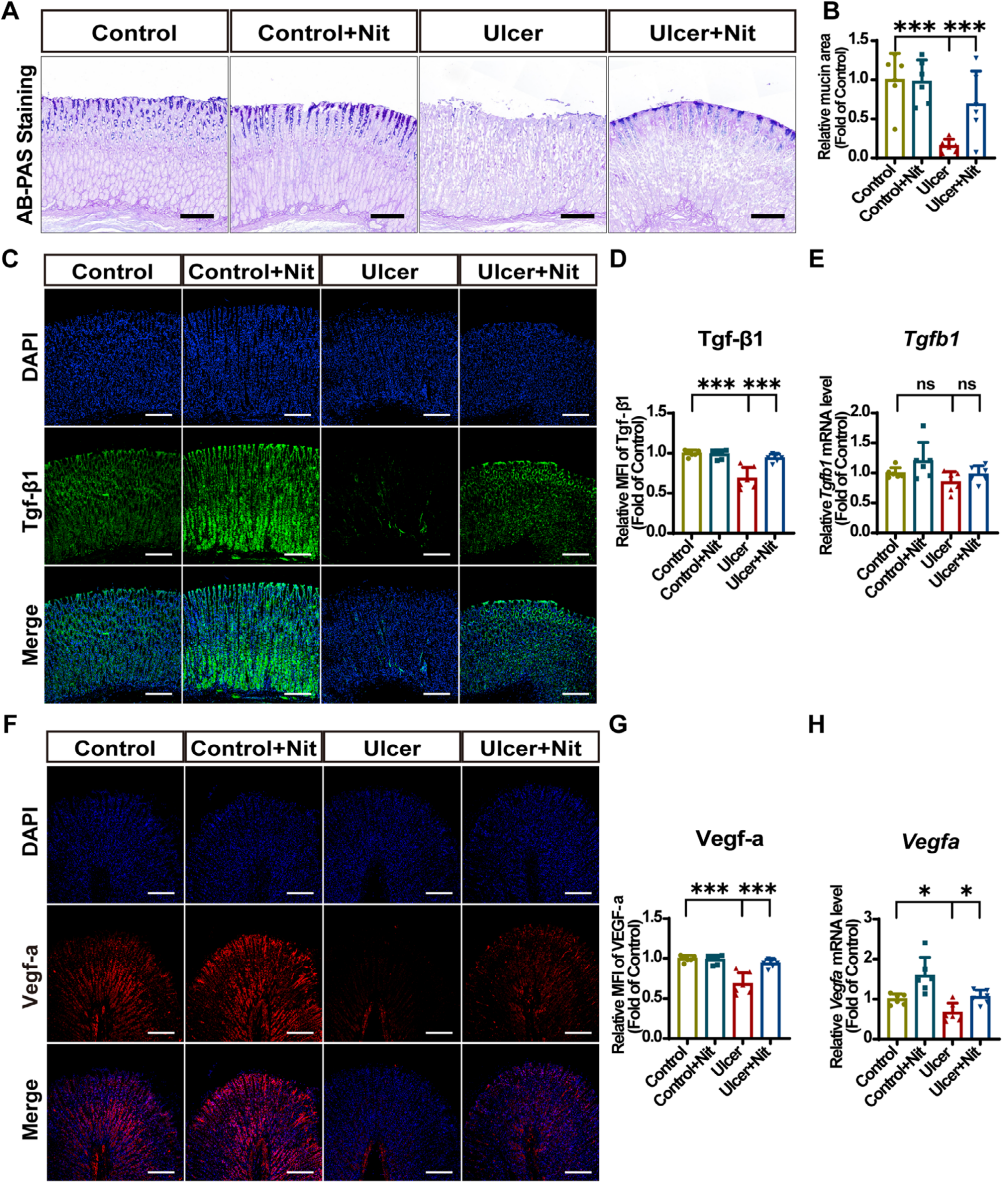
Dietary nitrate stabilizes the gastric mucus layer and improves mucosal healing of ethanol‐induced injury in vivo. (A) Representative gastric tissue images of AB–PAS staining. Scale bar = 50 µm. (B) Mucin histochemical analysis of gastric tissue. The mucin area was expressed as fold change relative to the control group. (C) IF staining of Tgf‐β1 (green) and DAPI (blue). (D) IF analysis of Tgf‐β1 with MFI. Scale bar = 200 µm. (E) RT‐qPCR analysis of *Tgfb1* mRNA expression in gastric mucosa. Target gene expression was normalized to *Gapdh* mRNA and expressed as fold change relative to the control group. (F) IF staining of Vegf‐a (red) and DAPI (blue). Scale bar = 200 µm. (G) IF analysis of Vegf‐a with MFI. (H) RT‐qPCR analysis of *Vegfa* mRNA expression in gastric mucosa. Target gene expression was normalized to *Gapdh* mRNA and expressed as fold change relative to the control group. Quantitative data are expressed as the mean ± SD. *p < 0.05, ***p < 0.001, and ns denotes no significance. IF, immunofluorescence; Nit, nitrate; RT‐qPCR, real‐time quantitative polymerase chain reaction; DAPI, 2‐(4‐amidinophenyl)‐6‐indolecarbamidine dihydrochloride; AB–PAS, alcian blue and periodic acid‐Schiff; Vegf‐a, vascular endothelial growth factor a; Tgf‐β1, transforming growth factor beta 1; MFI, mean fluorescence intensity; SD, standard deviation.

Certain alcoholic beverages are potent stimulators of gastrin release and gastric acid production, which even induce the maximum output of gastric acid [46]. Excessive gastric acid would erode the mucus layer. Alcian blue and periodic acid‐Schiff (AB–PAS) staining were used to observe gastric mucus thickness. In the control group, mucus layer above the gastric mucosa appeared dense and continuous, whereas in the ulcer group, the mucus layer was thinner and even showed signs of detachment. However, the ulcer + Nit group showed a statistical increase in mucus thickness (Figure 2A). Quantitative results (Figure 2B) also indicated that nitrate effectively reversed EtOH‐induced mucus barrier breakage.

Growth factors are crucial for ulcer healing. Thus, we investigated the expression of two growth factors closely associated with epithelial recovery. Transforming growth factor beta 1 (Tgf‐β1) is a multifunctional cytokine and key regulatory factor in wound healing that promotes tissue repair at different stages of GU healing [47]. Vascular endothelial growth factor a (Vegf‐a) is a crucial protective factor for the gastric mucosa, playing an essential role not only in the formation of new blood vessels, but also in the proliferation and migration of epithelial cells [48]. Through IF staining analysis, we found that the Tgf‐β1 content in the epithelial cells of the ulcer and ulcer + Nit group was significantly decreased and increased, respectively compared with the control group (Figure 2C,D). Further, *Tgfb1* expression was verified using real‐time quantitative polymerase chain reaction (RT‐qPCR) (Figure 2E). IF staining of Vegf‐a and RT‐qPCR for *Vegfa* exhibited a similar tendency (Figure 2F–H). These results suggest that nitrate improves the healing of EtOH‐induced GUs.

### 2.3 Dietary Nitrate Reduces EtOH‐Induced Inflammatory Responses In Vivo

**FIGURE 3:**
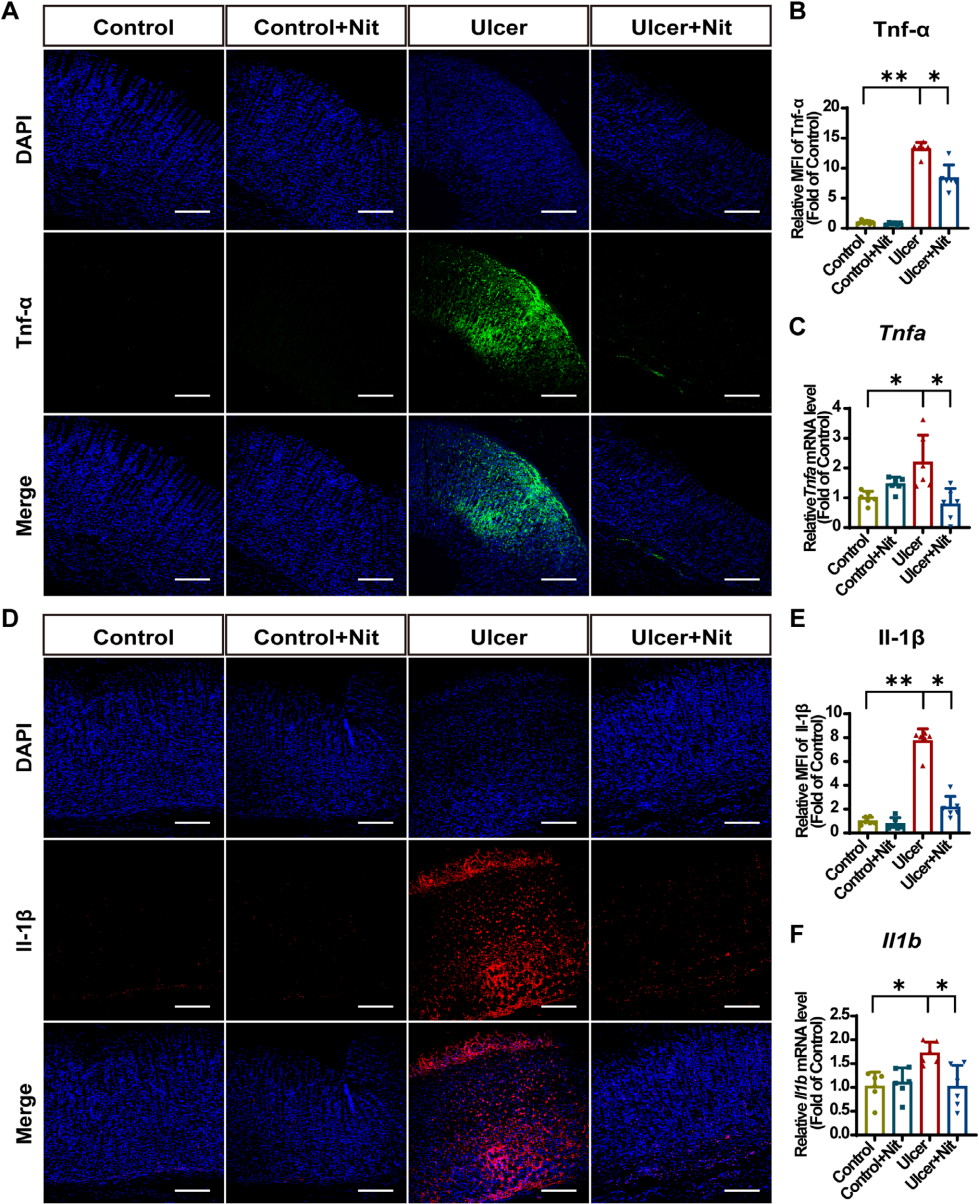
Dietary nitrate reduces ethanol‐induced inflammatory response in vivo. (A) IF staining of Tnf‐α (green) and DAPI (blue). (B) IF analysis of Tnf‐α with MFI. Scale bar = 200 µm. (C) RT‐qPCR analysis of *Tnfa* mRNA expression of the gastric mucosa. Target gene expression was normalized to *Gapdh* mRNA and expressed as fold change relative to the control group. (D) IF staining of Il‐1β (red) and DAPI (blue). Scale bar = 200 µm. (E) IF analysis of Il‐1β with MFI. (F) RT‐qPCR analysis of *Il1b* mRNA expression in gastric mucosa. The target gene expression was normalized to *Gapdh* mRNA and expressed as fold change relative to the control group. Quantitative data are expressed as the mean ± SD. *p < 0.05, **p < 0.01. IF, immunofluorescence; Nit, nitrate; RT‐qPCR, real‐time quantitative polymerase chain reaction; DAPI, 2‐(4‐amidinophenyl)‐6‐indolecarbamidine dihydrochloride; Tnf‐a, tumor necrosis factor alpha; Il‐1β, interleukin‐1β; MFI, mean fluorescence intensity; SD, standard deviation.

During GU development, tumor necrosis factor alpha (Tnf‐α) significantly increases, triggering an acute inflammatory response, inhibiting gastric microcirculation, and delaying ulcer healing [49]. In our study, Tnf‐α expression was evaluated using IF staining and RT‐qPCR (Figure 3A–C). The results demonstrated that compared with the control group, the ulcer group exhibited elevated Tnf‐α levels, whereas the ulcer + Nit group showed a significant decrease in Tnf‐α expression. *Tnfa* mRNA levels were consistent with the IF staining results.

Neutrophil infiltration of the gastric mucosa is a critical process in GUs’ pathogenesis. Interleukin‐1β (IL‐1β) secretion can promote the expression of adhesion molecules, including intercellular adhesion molecule 1 and vascular cell adhesion molecule 1, which are involved in infiltrating neutrophils in ulcerated tissues [50]. Il‐1β levels were detected using IF staining and RT‐qPCR, revealing a trend consistent with that of Tnf‐α (Figure 3D–F). EtOH significantly increased Il‐1β protein and *Il1b* mRNA expression in gastric mucosa, whereas nitrate supplementation significantly decreased these levels. Therefore, it can be inferred that nitrate reduces Tnf‐α and Il‐1β expression to exert anti‐inflammatory effects and promote ulcer healing.

### 2.4 Dietary Nitrate Upregulates Tff2

**FIGURE 4:**
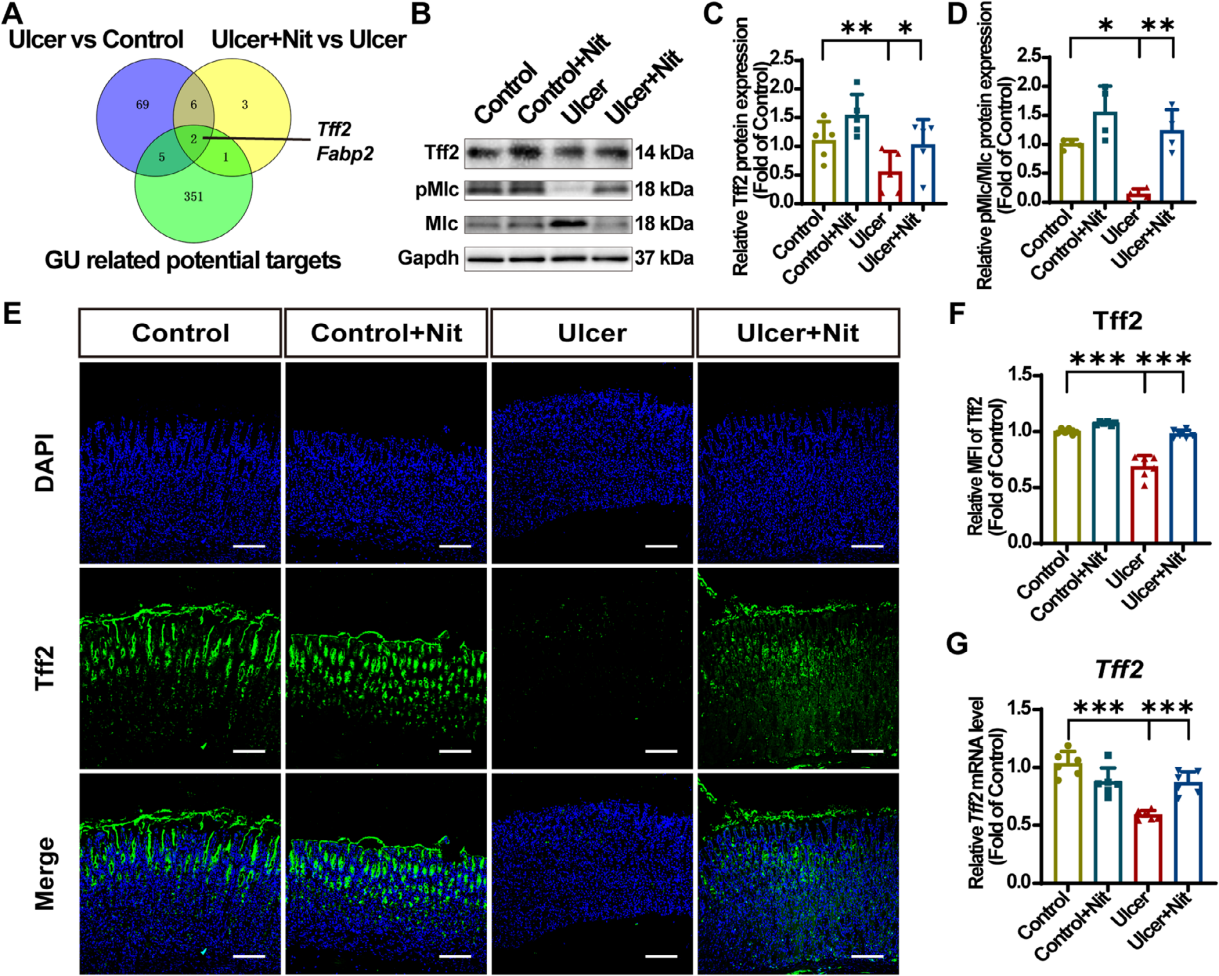
Dietary nitrate upregulates Tff2. (A) Venn diagram showing the unique and overlapping genes presented in paired groups and GU‐related potential targets. (B) Representative immunoblotting band of Tff2 and Mlc protein. (C and D) Analyses of immunoblotting band gray value in (B). (E and F) Representative Tff2 IF staining images in gastric mucosa and MFI score. Scale bar = 100 µm. (G) RT‐qPCR analysis of *Tff2* mRNA expression in gastric mucosa. Target gene expression was normalized to *Gapdh* mRNA and expressed as fold change relative to the control group. Quantitative data are expressed as the mean ± SD. *p < 0.05, **p < 0.01, ***p < 0.001. GU, gastric ulcer; Nit, nitrate; Mlc, myosin light chain 2; pMlc, phosphorylated myosin light chain 2; IF, immunofluorescence; RT‐qPCR, real‐time quantitative polymerase chain reaction; DAPI, 2‐(4‐amidinophenyl)‐6‐indolecarbamidine dihydrochloride; MFI, mean fluorescence intensity; Tff2; trefoil factor 2; SD, standard deviation.

To further explore the role of nitrate in protecting against EtOH‐induced GUs, differential gene expression analysis was performed using RNA sequencing (RNA‐seq). Set the standard as p‐adj <0.01 and an absolute fold change >5. The results revealed that 87 genes were downregulated by EtOH and 12 genes were upregulated by nitrate. Subsequently, an intersection was obtained from the previously studied GU‐related gene set [51], leading to the identification of two genes: fatty acid‐binding protein 2 (*Fabp2*) and *Tff2* (Figure 4A). FABP2 primarily participates in the transport, absorption, and metabolism of intestinal long‐chain fatty acids [52] with lower relevance to GUs. TFF2, initially considered a mucin constituent known as a luminal surveillance peptide (LSP), has a strong relationship with mucus stabilization and epithelial restitution [26]. Myosin light chain 2 (MLC) is a component of nonmuscle myosin II, and its phosphorylation has a positive effect on epithelial cell migration [53]. Within tissue, the phosphorylation of Mlc promotes re‐epithelialization by regulating the loosening and re‐establishment of intercellular adhesion junctions and tight junctions [54, 55]. Within the cell, phosphorylation of MLC stimulates cytoskeletal contraction and also helps cells maintain orientation and momentum during migration [56].

To further validate the critical role of Tff2 in nitrate protection against EtOH‐induced GUs, Tff2, Mlc, and phosphorylated Mlc (pMlc) protein levels in gastric tissues were examined. Consistent with the RNA‐seq data, EtOH exposure significantly decreased Tff2 expression in the ulcer group compared with that in the control group. Nitrate supplementation inhibited this decrease (Figure 4B–D), and IF staining and RT‐qPCR for Tff2 showed a similar trend (Figure 4E–G). MLC phosphorylation level was lower in the ulcer group than in the control group. Nitrate reversed this effect. The pMlc/Mlc ratio was significantly reversed between the groups, suggesting that nitrate upregulated Tff2 to promote gastric epithelial cell migration and activate mucosal recovery (Figure 4B–D).

### 2.5 Tff2 Knockdown Abolishes Nitrate Protective Effect Against EtOH‐Induced GUs In Vivo

**FIGURE 5:**
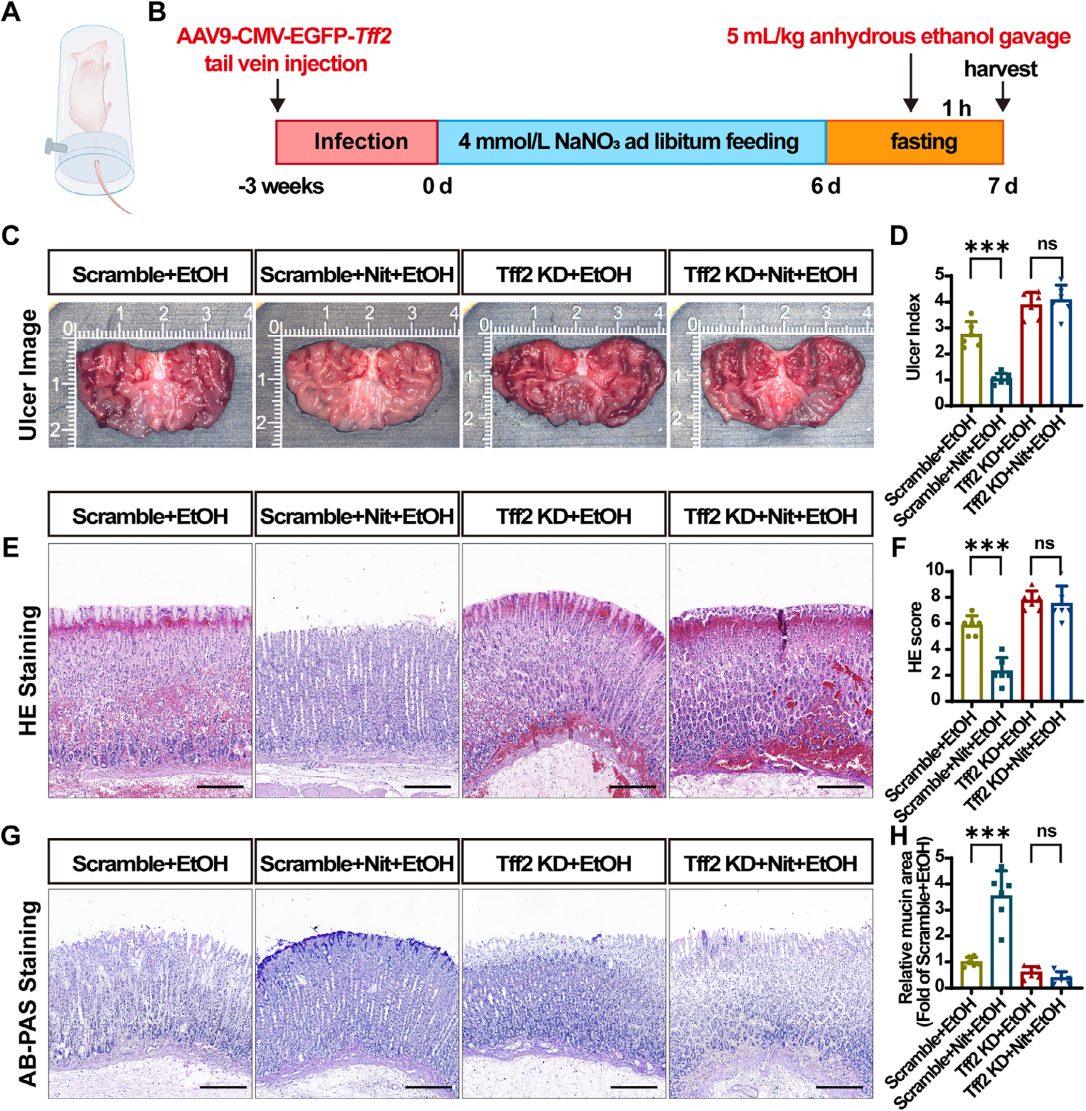
Tff2 knockdown abolishes nitrate protection against ethanol‐induced gastric ulcers in vivo. (A) Establishment of Tff2‐KD rat by tail vein injection of recombinant AAV. (B) The timeline of dietary nitrate administration (3 weeks after injection and 7 days before ethanol gavage). (C and D) The macroscopic appearance and ulcer index of the gastric mucosa in Tff2‐KD and scramble groups with ethanol gavage. The UI was calculated as follows: UI = 10 × (ulcerated area/total mucosal area). (E and F) Representative histology images of stomach tissue and a histopathologic score of HE staining in Tff2‐KD and scramble groups. The HE score sums the severity of four features: inflammatory cells (0–3), mucosal edema (0–4), hemorrhage (0–4), and epithelial loss (0–3). Scale bar = 50 µm. (G and H) Representative gastric tissue images of AB–PAS staining and mucin histochemical analysis of Tff2‐KD and scramble groups. The mucin area was expressed as fold change relative to the scramble + EtOH group. Scale bar = 50 µm. Quantitative data are expressed as the mean ± SD. ***p < 0.001, and ns denotes no significance. AAV, adeno‐associated virus; HE, hematoxylin–eosin; AB–PAS, Alcian blue and periodic acid‐Schiff; EtOH, ethanol; KD, knockdown; Nit, nitrate; Tff2; trefoil factor 2.

To investigate whether Tff2 is a key target of nitrate antiulcer effects, we established Tff2‐knockdown (KD) rats. Based on previous studies [57], we infected the whole body of Sprague–Dawley (SD) rats with recombinant adeno‐associated virus (AAV) vector serotype 9. Three‐week‐old male SD rats were transfected via tail vein injection with either an AAV–Tff2 gene KD vector (AAV–Tff2‐KD) or a negative control vector (AAV–NC) (Figure 5A,B). Transfection efficiency was validated by western blot and cryosectioning (Figure S1).

Consistent with the aforementioned findings, nitrate supplementation significantly alleviated gastric mucosal hemorrhage and oedema in the AAV–NC‐transfected group (scramble + EtOH/scramble + Nit + EtOH). However, nitrate protection diminished in the AAV–Tff2‐KD‐transfected group (Tff2‐KD + EtOH/Tff2‐KD + Nit + EtOH) (Figure 5C,D). Compared with the scramble + EtOH group, rats in the Tff2‐KD + EtOH group exhibited more severe erythrocytes infiltration and submucosal oedema, with nitrate supplementation showing no apparent effect (Figure 5E,F). Similarly, AB–PAS staining revealed that the mucus layer loss in the Tff2‐KD + EtOH group was greater than that in the scramble + EtOH group. Moreover, nitrate did not ameliorate this alteration (Figure 5G,H). These findings indicated that GUs were more severe in the Tff2‐KD + EtOH group than in the scramble + EtOH group, and nitrate protection was significantly reduced. Nitrate treatment also affects inflammatory levels and epithelial barrier function (Figure S2). Thus, we determined that Tff2 is the key molecule that nitrate employs to protect gastric mucosa against EtOH.

### 2.6 Nitrate Promotes Migration by TFF2 Upregulation in vitro

**FIGURE 6:**
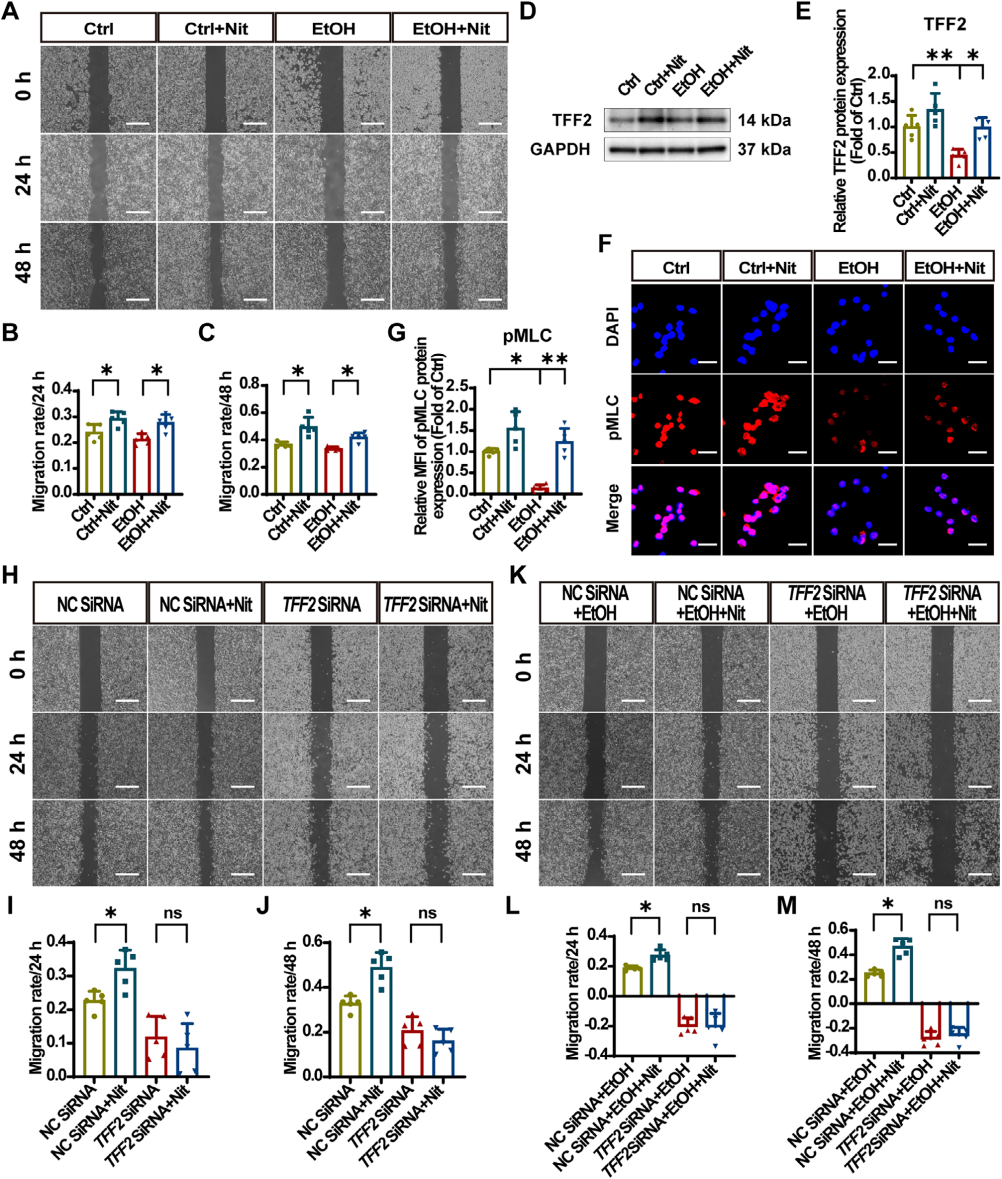
Nitrate promotes migration by TFF2 upregulation in vitro. (A) Images of the scratch healing process of GES‐1 cells in Ibidi culture inserts. Scale bar = 500 µm. (B and C) Quantitative analysis of the migration rate at 24 h and 48 h in (A). (D and E) Representative immunoblotting band of the TFF2 protein and analysis of band gray values. (F) IF staining of pMLC (pink) and DAPI (blue). Scale bar = 40 µm. (G) IF analysis of pMLC with MFI. (H) Images of the scratch healing process of GES‐1 cells transfected with si‐*TFF2*/si‐negative control in Ibidi culture‐inserts. Scale bar = 500 µm. (I and J) Quantitative analysis of the migration rate at 24 h and 48 h. (K) Images of the scratch healing process of GES‐1 cells transfected with si‐*TFF2*/si‐negative control in Ibidi culture‐inserts containing ethanol. Scale bar = 500 µm. (L and M) Quantitative analysis of the migration rate at 24 h and 48 h. Migration rate is quantified by the percentage of closed area to the initial scratch area. Quantitative data are expressed as the mean ± SD. *p < 0.05, **p < 0.01, and ns denotes no significance. TFF2, trefoil factor 2; GES‐1, human gastric epithelial; EtOH, ethanol; Nit, nitrate; Ctrl, control; MLC, myosin light chain 2; pMLC, phosphorylated myosin light chain 2; DAPI, 2‐(4‐amidinophenyl)‐6‐indolecarbamidine dihydrochloride; NC, negative control; SD, standard deviation.

To further validate the effect of nitrate on EtOH‐induced epithelial barrier damage, we cultured human gastric epithelial (GES‐1) cells in vitro and conducted wound healing assays following EtOH stimulation to determine whether nitrate pretreatment enhances migration. GES‐1 cells are commonly employed for in vitro models of EtOH‐induced gastric mucosal injury [58, 59, 60, 61]. The results indicated that the control group exhibited significant migratory ability at 24 h and 48 h postscratch, with the control + Nit group demonstrating significantly stronger migration. However, the migratory repair capacity decreased after EtOH stimulation. Notably, nitrate supplementation significantly restored migration rates of GES‐1 cells (Figure 6A–C). To explore the therapeutic effect of nitrate, in the same scratch healing assay, nitrate was administered after EtOH stimulation. It was found that the ability of nitrate to promote the migration of GES‐1 cells was comparable to that of nitrate administered in advance (Figure S3A–C). Meanwhile, the promoting migration ability of nitrate and the expression of key genes were also verified in primary human gastric mucosal epithelial (CP‐H048) cells (Figure S3D–I).

To investigate TFF2's role in nitrate‐induced cell migration, we assessed TFF2 expression in GES‐1 cells. EtOH stimulation reduced TFF2 expression, whereas nitrate treatment significantly enhanced TFF2 levels (Figure 6D,E). In addition, it increased pMLC expression (Figure 6F,G). Furthermore, *TFF2*‐KD in GES‐1 cells significantly decreased migration, which was much slower compare with the NC groups (Figure 6H–J). Following EtOH stimulation, the migration rate became negative in the *TFF*2 small interfering RNA (siRNA) group, and nitrate was unable to rescue this decrease (Figure 6K–M). Transfection efficiency was validated by RT‐qPCR (Figure S4A).

In conclusion, we demonstrated that nitrate promotes migratory repair by enhancing GES‐1 cells motility and that TFF2 plays a critical role in this process in vitro.

### 2.7 Dietary Nitrate Inhibits the Notch Pathway in EtOH‐Induced GU

**FIGURE 7:**
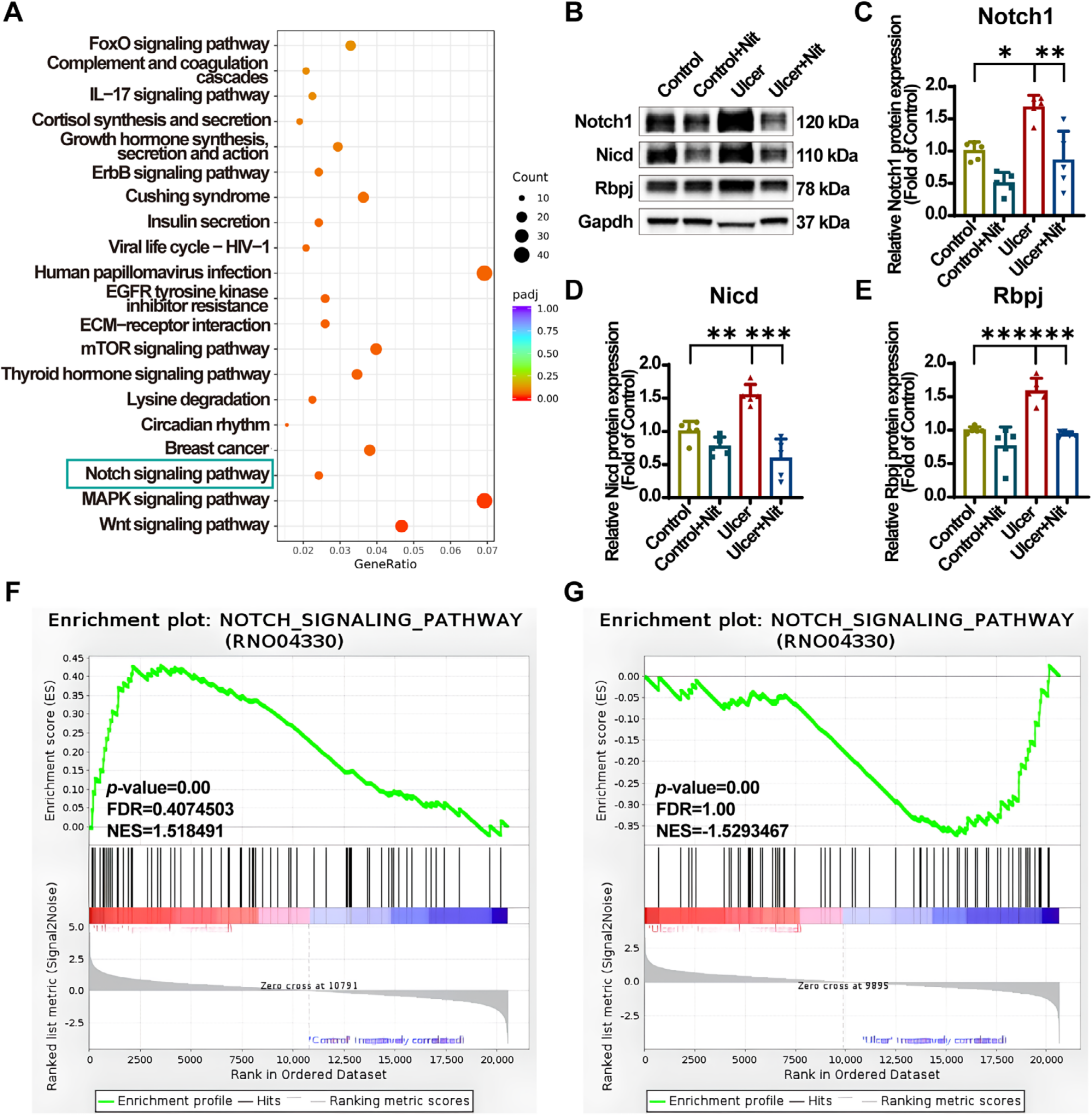
Dietary nitrate inhibits the Notch pathway overactivated by ethanol in SD rats. (A) Bubble plot showing the top 20 significantly enriched KEGG pathways. The abscissa is the ratio of the number of differential genes annotated to the KEGG pathway to the total number of differential genes, and the ordinate is the KEGG pathway. (B) Representative immunoblotting band of Notch1, Nicd, and Rbpj protein in gastric mucosal tissue. (C–E) Analyses of immunoblotting band gray value in (B). (F) GSEA analysis of Notch signaling pathway between ulcer and control groups. (G) GSEA analysis of Notch signaling pathway between ulcer + Nit and ulcer groups. Quantitative data are expressed as the mean ± SD. *p < 0.05, **p < 0.01, ***p < 0.001. KEGG, Kyoto Encyclopedia of Genes and Genomes; Nicd, intracellular structural domain; Rbpj, recombination signal binding protein for immunoglobulin kappa J region; GSEA, gene set enrichment analysis; RNA seq, RNA sequencing; SD,D standard deviation.

To elucidate the underlying mechanisms, a Kyoto Encyclopedia of Genes and Genomes (KEGG) pathway enrichment analysis was conducted. A comparison was performed using KEGG enrichment analysis to determine the top 20 significantly changed pathways between the ulcer and control groups, as well as between the ulcer + Nit and ulcer groups. Notch signaling pathway was ranked in the ulcer versus control groups as top 7, and ulcer + Nit versus ulcer groups as top 3 (Figure 7A). Notch signaling pathway was significantly activated in the ulcer group compared with the control group and significantly downregulated in ulcer + Nit given nitrate. The above trend was not seen in the other pathways that were enriched, and as the Notch signaling pathway is closely related to cell movement and migration [62, 63, 64], further experiments were designed to validate the correlation.

To further validate this finding, western blot analysis was conducted to assess key protein expression in the Notch pathway in gastric mucosa, aligned with RNA‐seq results. When the Notch1 receptor is activated, its intracellular structural domain (Nicd) is released from the cytoplasmic membrane and translocates to the nucleus, where it forms a complex with the transcription factor Rbpj (recombination signal binding protein for immunoglobulin kappa J region) and the coactivator Maml1 (Mastermind‐like 1) to regulate downstream target genes [64]. Compared with the control group, EtOH exposure significantly increased Notch1, Nicd (signaling messenger), and Rbpj (DNA‐binding protein) expression in rats, whereas nitrate administration reversed this increase (Figure 7B–E). Gene set enrichment analysis (GSEA) also indicated that Notch pathway‐related genes were upregulated in ulcerated tissues but downregulated in nitrate‐treated tissues (Figure 7F,G).

In summary, we discovered that EtOH overactivates the Notch pathway, whereas nitrate preadministration downregulates it, potentially playing a role in nitrate gastroprotection.

### 2.8 Nitrate Functions Positively Transcripts TFF2 by Notch Pathway Inhibition in vitro

To further investigate the upstream mechanism of TFF2 regulation, we utilized the JASPAR database (http://jaspar.genereg.net) and the Human transcription factor database (Human TFDB) to predict transcription factors with binding potential to the TFF2 promoter. The transcription factors with high correlation to the expression of TFF2 in gastric mucosa and play crucial roles in top enriched KEGG pathways were preliminarily inspected by RT‐qPCR (Figure S4B–O). Among these, RBPJ showed the most significant trend.

**FIGURE 8:**
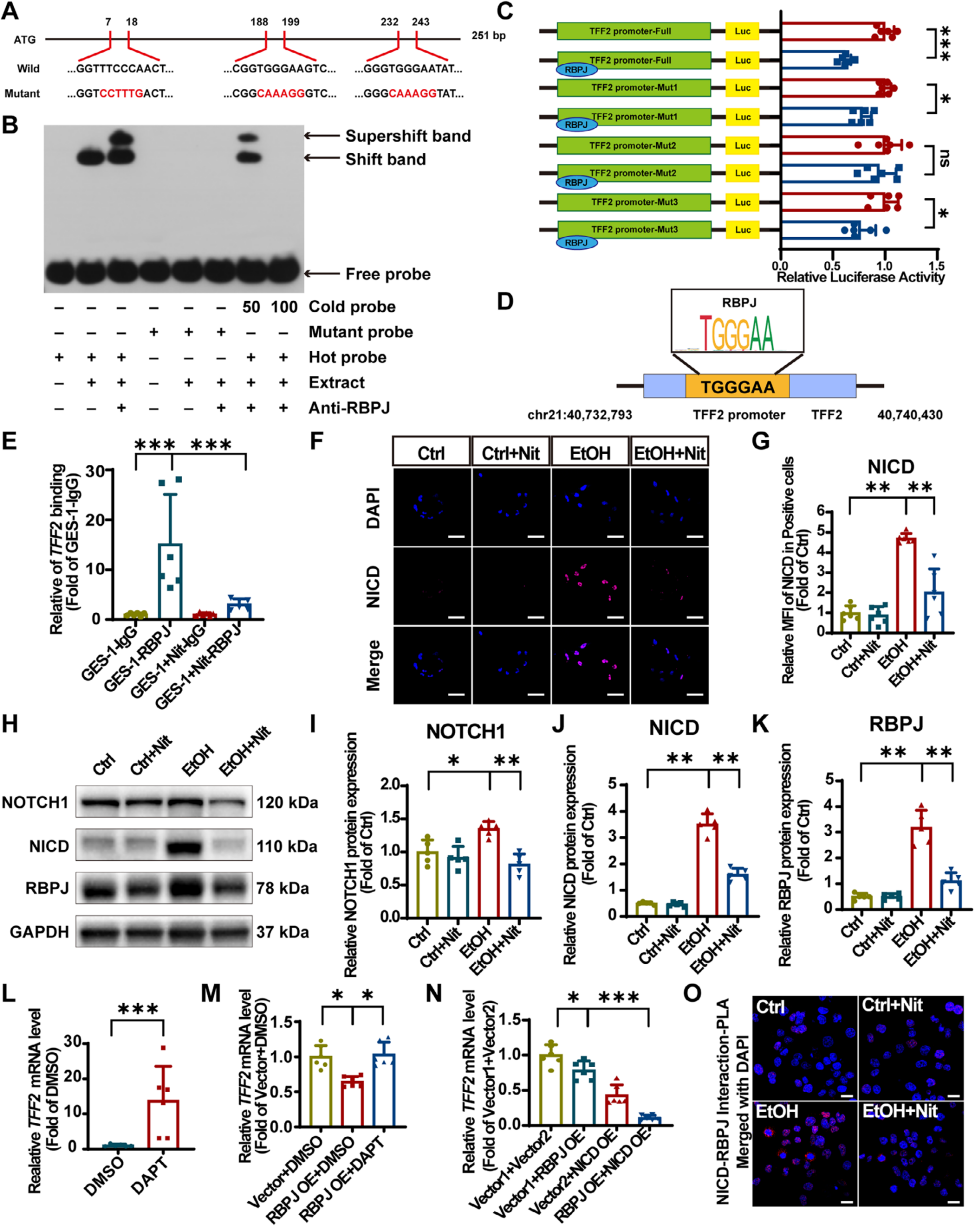
Nitrate functions by Notch pathway inhibition positively transcripting TFF2 in vitro. (A) Diagram of the wild‐type and mutant sequences for the three predicted RBPJ binding sites within the 251 bp TFF2 promoter probes. (B) Representative electrophoretic mobility shift assay (EMSA) autoradiograph. Hot probe is the biotin‐labeled wild‐type oligonucleotides of the truncated TFF2 promoter containing the binding motif; Mutant probe is the labeled oligonucleotides sequence with nucleotides mutated. The cold probe is nonlabeled competitive wild‐type probes (100 and 50 that of the concentrations). The shifted bands are indicated by arrows, which suggested the formation of DNA–protein complexes (lane 2, 3, 7). The super shifted bands indicated the formation of DNA–protein–antibody complexes (lane 3, 7). “+” and “−” represent presence and absence, respectively. (C) Relative TFF2 promoter (Full, Mut1, Mut2, and Mut3) luciferase activity was detected by DLR assays in RBPJ overexpressed and normal‐expressed GES‐1 cells. (D) A schematic diagram showing the location of RBPJ putative binding regions on the TFF2 promoter. (E) RT‐qPCR analysis of *TFF2* binding site expression of GES‐1 cells. Target site expression in RBPJ‐treated groups was normalized to IgG negative control groups and expressed as fold change relative to the IgG groups. (F) IF staining of NICD (pink) and DAPI (blue). Scale bar = 50 µm. (G) IF analysis of MFI of nuclear NICD in positive cells. (H) Representative immunoblotting band of Notch signaling pathway in GES‐1 cells. (I–K) Analyses of immunoblotting band gray value of (H). (L) RT‐qPCR analysis of *TFF2* mRNA expression of DMSO/DAPT treated GES‐1 cells. Target gene expression was normalized to *GAPDH* mRNA and expressed as fold change relative to the DMSO vehicle group. (M) RT‐qPCR analysis of *TFF2* mRNA expression in RBPJ overexpressed and NICD deprived GES‐1 cells. The target gene expression was normalized to *GAPDH* mRNA and expressed as fold change relative to the vector + DMSO group. (N) RT‐qPCR analysis of *TFF2* mRNA expression in RBPJ overexpressed and NICD‐RBPJ overexpressed GES‐1 cells. The target gene expression was normalized to *GAPDH* mRNA and expressed as fold change relative to the vector1 + vector2 group. (O) Representative image of PLA of NICD–RBPJ proximity. Each red dot represents a positive signal of NICD–RBPJ interaction and nuclei were counterstained with DAPI (blue). Scale bar = 20 µm. Quantitative data are expressed as the mean ± SD. *p < 0.05, **p < 0.01, ***p < 0.001, and ns denotes no significance. Nit, nitrate; EtOH, ethanol; GES‐1, human gastric epithelial; NICD, intracellular structural domain; RBPJ, recombination signal binding protein for immunoglobulin kappa J region; EMSA, electrophoretic mobility shift assay; TFF2, trefoil factor 2; DAPI, 2‐(4‐amidinophenyl)‐6‐indolecarbamidine dihydrochloride; Yhhu‐3792, N2‐(4‐isopropylphenyl)‐5‐(3‐methoxyphenoxy) quinazoline‐2,4‐diamine; Luc, luciferase; SD, standard deviation; DLR, dual‐luciferase report; PLA, proximity ligation assay.

To explore whether RBPJ play a physical interaction or functional binding to the TFF2 promoter, electrophoretic mobility shift assay (EMSA) and dual‐luciferase reporter assay (DLR) were conducted. The three mutated sites are shown in the Figure 8A. In the lane added with the RBPJ antibody and extract of GES‐1 cells, a super shift migration band showed that RBPJ transcription factors can directly bind to the TFF2 promoter. However, the mutant probe with mutated binding sites could not bind to the protein in the extract of GES‐1 cells. This indicates that RBPJ specifically interacts with the three predicted sites in the TFF2 promoter. The construction of the DNA–TFF2 complex is specific because only the excess cold (unlabeled wild‐type) probe can block it (Figure 8B). Moreover, the DLR results indicated that RBPJ overexpression slightly decreased luciferase activity. The mutation at the Mut2 (−1643 to −1638 bp) binding site did not lead to a significant reduction in promoter activity, while there was no significant difference between the Full and Mut1, Mut3 (Figure 8C). The Mut1, Mut2, and Mut3 plasmids, which correspond to the highest‐scoring transcription factor binding sites (TFBSs), were mutated separately (Figure S5). This indicates that TGGGAA is the binding site of RBPJ in the TFF2 promoter and is indispensable for RBPJ to trigger TFF2 transcriptional inhibition (Figure 8D). These data suggest that RBPJ can directly be bound to the TFF2 promoter and inhibits TFF2 transcriptional initiation. To elucidate the role nitrate plays in the RBPJ–TFF2 promoter bonding, CUT&Tag qPCR analysis was performed in GES‐1 cells. It shows nitrate downregulated the expression of these binding sites (Figure 8E). In summary, our findings suggest that RBPJ repressed transcription of TFF2. Nitrate might diminish the repression by decreasing the bounding of RBPJ to TFF2 promoter.

As observed in vivo, the Notch signaling pathway was activated by EtOH and downregulated by nitrate. Given that RBPJ is a crucial transcription factor of Notch signaling pathway, we strongly suspect that nitrate may regulate TFF2 expression by the Notch pathway. IF staining revealed that NICD was abundantly translocated into the cell nucleus following EtOH stimulation, which was alleviated by nitrate (Figure 8F,G). Western blot result showed that EtOH stimulation significantly increased NOTCH1, NICD, and RBPJ protein expression of GES‐1 cells, whereas nitrate markedly downregulated these expressions (Figure 8H–K). Furthermore, Notch pathway activation in GES‐1 cells using Yhhu‐3792 significantly decreased cell migration, as observed in wound healing assays. Notch pathway activation efficiency was validated by RT‐qPCR (Figure S6A–D). After EtOH stimulation, cells with an activated Notch pathway showed minimal migration, with nitrate showing no protection (Figure S6E–G).

Since EtOH can overactivate the Notch signaling pathway, leading to RBPJ overexpression and a large amount of NICD entering the nucleus, while nitrate can inhibit both, to observe whether NICD plays a role in the process of RBPJ inhibiting TFF2 transcription, we conducted an NICD deprivation experiment. While RBPJ overexpression significantly suppressed TFF2 expression, pharmacological inhibition of Notch cleavage using DAPT, which deprives the nucleus of NICD, completely reversed this inhibitory effect (Figures 8L–M). To explore who dominates the transcriptional regulation of TFF2, we compared the effect of RBPJ/NICD overexpression on *TFF2* mRNA level. As shown in the figure, we found that the simultaneous overexpression of NICD and RBPJ had a much greater weakening effect on *TFF2* abundance than the overexpression of RBPJ alone (Figures 8N). The NICD deprivation efficiency of DAPT and the overexpression efficiency of the NICD overexpression plasmid and RBPJ overexpression plasmid are presented in the Figure S6H–L. Next, we performed in situ proximity ligation assay (PLA) between NICD and RBPJ in nitrate and EtOH treated GES‐1 cells. Very few PLA puncta were observed when the Notch signaling pathway was not overactivated by EtOH in GES‐1 cells. Abundant PLA signal (red dots) was observed in EtOH group than control group, indicating an increase of NICD‐RBPJ complex formation. A significant decrease was found in the amount of PLA puncta in EtOH + Nit group compared with EtOH group (Figures 8O). Based on the above results, we draw the following conclusion: under the background of excessive activation of the Notch signaling pathway by EtOH, nitrate inhibits NICD from entering the nucleus, reduces the formation of the NICD–RBPJ complex, leads to a decrease in the binding of the transcription factor RBPJ to the TFF2 promoter, partially relieves transcriptional inhibition, and results in an increase in TFF2 expression.

## 3 Discussion

The innovative findings of this study suggest that nitrate enhances gastric mucosal defense against EtOH stimulation and attenuates mucosal injury by stabilizing the mucus layer, enhancing the vascular endothelial barrier and accelerate the restitution process of mucosal epithelial cells. In this paper, we also verified the function of nitrate in increasing mucosal blood supply and thickening the gastric mucus layer as reported in previous literature [41]. Previous studies have attributed the protective effects of nitrate to the functions of vasodilatory and progastric mucus secretory effects of NO [42, 44]. However, we believe that nitrate is more than a mere exogenous NO donor and that there probably be more valuable modes of regulation in addition to the NO pathway. Our results suggest that nitrate promotes *TFF2* transcription by downregulating the EtOH‐overactivated Notch pathway, enhancing gastric mucosa defense and repair process in EtOH‐induced gastric mucosal injury.

TFF2 is an important gastric LSP discovered in recent years. LSPs are a class of peptides that play crucial roles in the gastric mucosa by sensing environmental changes in the gastric lumen, such as pH, osmotic pressure, and microbes, and regulating gastric mucosa defense and repair through specific pathways [26]. TFF2 exerts diverse beneficial effects on the gastric mucosa. It acts not only as a mucin peptide enhancing mucus barrier by increasing mucin hydrophobicity, but also initiates early gastric mucosa repair [24, 65]. After transcriptome analysis predicted *Tff2* as a key target for nitrate to exert gastric mucosal protective effects, Tff2‐KD rats and *TFF2*‐KD cells were established. It was found that the protective effect of nitrate on the strength of barriers and the promotion of cell migration was abolished. Thus far, we concluded that *Tff2* is a key nitrate target for enhancing gastric mucosal defense and promoting early epithelial repair.

Since the upstream regulatory mechanism of TFF2 has not been fully elucidated [66]. To elucidate how nitrate regulates TFF2, we explored it through data analysis and a series of experiments. Sequencing and enrichment analysis predicted that the Notch signaling pathway may play an important role in this model. Following EtOH exposure, Notch pathway activation was observed, which was downregulated by nitrate. The Notch signaling pathway is a highly conserved pathway for cell fate decisions closely related to cell movement regulation [67]. Through a classical three‐step cleavage process, active NICD is released from the transmembrane NOTCH receptor into the cytoplasm. Upon translocation to the nucleus, NICD binds to the crucial NOTCH transcription factor RBPJ (usually associated with a corepressor and acts to repress Notch target genes), forming the NICD–RBPJ–MAML complex that regulates the transcription of Notch target genes [63, 68, 69, 70, 71]. Our results suggest that nitrate, by downregulating the overactivated Notch pathway by EtOH, negatively regulates NICD–RBPJ complex to promote *TFF2* transcription, increases TFF2 expression, improves the gastric mucosal barriers, facilitates gastric epithelial cell migration, and promotes the defense and repair of EtOH‐induced GUs, thus offering new strategies for clinical prevention.

Although EtOH‐induced GU in rats share pathological and histological similarities with human GUs [72], and EtOH exposure models mimic human binge drinking, animal models cannot fully replicate the complex pathology of human GUs. To enhance clinical relevance, we standardized pathogenic conditions to ensure lesion severity closely resembled human EtOH‐induced damage. Additionally, two human gastric mucosal epithelial cell lines (GES‐1 and CP‐H048) were used to evaluate nitrate's effect on epithelial repair, partially compensating for the lack of human data. These findings provide a theoretical basis for the clinical application of nitrate. To maintain stable blood nitrate levels, rats received 4 mmol/L sodium nitrate in drinking water for 7 days before EtOH stimulation. While prolonged preadministration has clinical constraints, single high‐dose gavage could irritate the mucosa. Moreover, nitrate's therapeutic effect postulcer formation was not examined. Our team is developing sustained‐release nitrate nanoparticles [37] to shorten onset time, extend duration, and reduce mucosal irritation. These advances may address current limitations. Based on our results and prior studies [42, 73], nitrate shows promise as a dietary supplement for high‐risk individuals (e.g., those who consume alcohol or take NSAIDs) or as an adjuvant to conventional antiulcer drugs. Ongoing research on its mechanisms and formulations supports its huge potential for clinical translation in ulcer prevention and treatment.

## 4 Materials and Methods

### 4.1 Animals

Charles River Laboratories (Beijing, China) supplied 6‐week‐old male SD rats weighing 180 ± 20 g. All animals were housed in the Capital Medical University Laboratory Animal Centre on a 12‐h light/dark cycle at 24.0 ± 1.0°C and 55–65% relative humidity in a particular pathogen‐free environment. Sufficient rodent food and water were provided around the clock. The Capital Medical University Institutional Animal Care and Use Committee gave its approval to all procedures (Ethical Approval: AEEI‐2023‐110).

After 12‐h adaptation, 24 SD rats were randomized into four groups. Two of the four groups were administered 4 mmol/L sodium nitrate in their drinking water. The other two groups continued to receive double distilled water. On the 6th day, all animals were fasted and restrained with water for 24 h. On the 7th day, an EtOH‐induced GU model was established by gavage with anhydrous EtOH (5 mL/kg). The SD rats were divided into the following four groups (*n* = 6/group):

1. Control group: ordinary drinking water and distilled water gavage;
2. Control + Nit group: 4 mmol/L sodium nitrate drinking water and distilled water gavage;
3. Ulcer group: ordinary drinking water and anhydrous EtOH gavage;
4. Ulcer + Nit group: 4 mmol/L sodium nitrate drinking water and anhydrous EtOH gavage.

On the 7th day, 1 h after the instillation of anhydrous EtOH/distilled water, the serum and gastric tissues were collected immediately after anesthesia. The stomach was cut along the great curvature, and the chyme and surface mucus were rinsed with PBS precooled at 4°C. The abdomen of each rat was rapidly removed and opened along the larger curvature, glandular stomach tissues were visually examined for macroscopic analysis of stomach damage (measured as the ulcer index), and each abdomen was divided, and a half was submerged in a 4% paraformaldehyde‐fixed solution (G1101; PFA; Servicebio, Wuhan, China) for histopathological analyses. After separation of gastric mucosa layer, the other half was quick‐freezed by liquid nitrogen stored at a −80°C freezer for biochemical analyses.

### 4.2 Macroscopic Observation

After opening and rinsing, the stomachs were gently flattened to avoid stretching and to assess the gastric lesion index.

The flattened luminal side of the gastric samples were photographed by a digital stereo 3D full‐high‐definition inspection system (DRV‐Z1; Vision Engineering, UK). Image J 1.36b image analysis software (National Institutes of Health, Bethesda, MD, USA) was used to measure ulcerated area. The ulcer index was acquired as presented by Ganguly [74], using the equation where *X* is the total mucosal area/ulcerated area.

Ulcer index (UI) = 10/*X*.

### 4.3 Microscopic Examination

Gastric tissue samples were fixed in 4% PFA for 48 h, dehydrated with gradient EtOH and encased with paraffin. Subsequently, the stomach tissue was cut into pieces of approximately 5 µm thickness. After xylene dewaxing and gradient EtOH to water conversion, two slides were prepared for each sample, stained with HE solution, and then graded and assessed by two separate operators in a double‐blind manner employing a double‐blind technique. Three histological areas were examined for changes.

In accordance with the criteria outlined by Laine and Weinstein, the degree of microscopic gastrointestinal injury was quantified on a 0–14 scale. In summary, each histological section was analyzed for the presence of inflammatory cells (score: 0–3), upper mucosal oedema (score: 0–4), hemorrhagic damage (score: 0–4), and epithelial cell loss (score: 0–3) in a 1‐cm segment. The microscopic HE scores were calculated by adding the four histopathological scores. Two other slides were stained with AB–PAS to analyses mucus barrier damage. AB–PAS‐stained sections were used to assess neutral mucin content. Digital images of the gastric mucosa from standardized regions of identical length were acquired by an automated slide scanning system (Pannoramic SCAN; 3DHISTECH, Spain) provided by the Core Facility Center, Capital Medical University. The area of AB–PAS‐positive staining was quantified using ImageJ software and normalized to the control group's mean value for statistical comparison.

### 4.4 Immunostaining

For immunostaining, as with HE and AB–PAS, the slides were repaired with antigen in an antigen repair buffer, then 1 h of 1% goat serum to block antigens.

Next, we incubated the tissues with anti‐CD31 (1:200 dilution), anti‐TFF2 (1:500 dilution), anti‐Vegf‐a (1:1000 dilution), anti‐Tgf‐β1 (1:1000 dilution), anti‐Tnf‐α (1:500 dilution), and anti‐Il‐1β (1:500 dilution) antibodies at 4°C overnight and incubated with secondary antibodies (1:1000 dilution) for 1 h at room temperature. Slices were incubated with 2 µg/mL DAPI (C0060; Servicebio) to stain nuclei, followed by extensive washing with 1× Tris buffered saline with Tween 20. Fluorescence microscopy images were captured using confocal microscopy (AX NIS‐Elements 5.4; Nikon, Japan), and positive areas were counted using ImageJ software. Table S1 lists the antibodies that were employed.

### 4.5 RT‐qPCR

In accordance with the RNA extraction kit's instructions (DP431; Tiangen, Beijing, China), total RNA was isolated from samples of rat gastric epithelial tissue and the human gastric epithelial cell lines GES‐1 and CP‐H048. Reverse transcription of 2 µg RNA to cDNA using a PrimeScript RT kit (RR036A; Takara, Tokyo, Japan). Subsequently, cDNA was amplified by RT‐qPCR using the NovoStartSYBR qPCR SuperMix Plus (E096, novoprotein; Suzhou, China). Table S2 lists the gene primer sequences. Gene transcription levels were calculated using the 2^−∆∆C^ method.

### 4.6 Nitrate Level in Serum and Gastric Mucosa

The ultrafiltrate of serum and gastric mucosal tissues homogenate were collected. Different samples were diluted at different ratios and then tested. Nitrate concentration was detected using the total nitric oxide and nitrate/nitrite parameter assay kit (KGE001; R&D Systems, Minneapolis, MN, USA) per manufacturer instructions.

### 4.7 RNA Sequencing

RNA‐seq and enrichment analyses of gastric mucosa tissue were performed by Novogene (Beijing, China). We used cluster Profiler R package to test the statistical enrichment of differential expression genes in KEGG pathways. The Reactome database brings together the various reactions and biological pathways of human model species. Reactome pathways with corrected *p* value less than 0.05 were considered significantly enriched by differential expressed genes. Total RNA‐seq data were obtained from China National GeneBank Sequence Archive (CNSA) with accession number CNP0007543.

Differentially expressed genes were screened to identify the key nitrate targets. A corrected padj of 0.01 and an absolute fold change of 5 were established as criteria. Significantly enriched KEGG pathways were analyzed to identify the mechanisms of nitrate action.

### 4.8 Western Blot

Gastric mucosa tissue (30 mg/sample) was ground using a grinder (JXFSTPRP‐CL‐BSC; Jingxin, Shanghai, China). All samples were lysed with lysis buffer containing 1% phosphatase inhibitor (1260; Applygen, Beijing, China) and 0.5% protease inhibitor cocktail (539134; Merck, Germany). The BCA method was used to determine the total protein concentration. Proteins were denatured and separated by gel electrophoresis followed by PVDF membrane transfer. The membranes were incubated with primary antibodies against Tff2 (1:1000 dilution), Notch1 (1:1000 dilution), Nicd (1:1000 dilution), Rbpj (1:1000 dilution), Mlc (1:1000 dilution), pMlc (1:1000 dilution), Gapdh (1:4000 dilution) overnight at 4°C, and then probed with secondary antibodies conjugated to horse radish peroxidase (1:2000 dilution). The primary antibodies are listed in Table S3.

### 4.9 Tff2‐KD by AAV Infection

Twenty‐four male SD rats (3‐week‐old) were purchased from Charles River Laboratories (Beijing, China). They were also housed in the Capital Medical University Laboratory Animal Centre on the same conditions as above described. After 12‐h adaptation, 24 SD rats were randomized into two groups. The short hairpin RNA targeting *Tff2* was constructed based on the AAV9–CMV–MCS–3Flag–FT2A–EGFP–WPRE–BGH poly A precursor skeleton (GENECHEM Biotech, Shanghai, China). In Tff2‐KD group, 12 SD rats were injected with AAV9–Tff2‐KD (1 × 10^12^ µg per rat) via the tail veins to generate a Tff2‐KD rat model. In scramble group, the rest 12 SD rats were injected with the same amount of vector virus (AAV–NC) in the tail vein. Considering AAV transfection efficiency in rats, nitrate supplementation was introduced 3 weeks after the virus intervention. Then half of the rats in each group were given normal drinking water, and half were given 4 mmol/L nitrate drinking water. One week later, anhydrous EtOH was administered by gavage to all rats. The rats were divided into four groups based on tail vein injection and water intake as following (*n* = 6/group):

1. Scramble + EtOH group: AAV–NC injection and ordinary drinking water;
2. Scramble + Nit + EtOH group: AAV–NC injection and 4 mmol/L sodium nitrate drinking water;
3. Tff2‐KD + EtOH group: AAV–Tff2‐KD injection and ordinary drinking water;
4. Tff2‐KD + Nit + EtOH group: AAV–Tff2‐KD injection and 4 mmol/L sodium nitrate drinking water.

Considering AAV transfection efficiency in rats, nitrate supplementation was introduced 3 weeks after the virus intervention. Absolute EtOH was administered by gavage 1 week later. The animals were then euthanized. Serum and gastric tissue samples were collected and processed in the same way as uninfected SD rats (with no AAV intervention). The stomachs were divided into three parts after being photographed. The section prepared for histopathological analysis was handled as described above. HE, AB–PAS staining and immunostaining were conducted using the same protocol. Specimens of the second part were fixed in 4% PFA overnight at 4°C, rinsed with PBS, dehydrated in 30% sucrose solution (Macklin, Shanghai, China) at 4°C, excess solution around the tissues was wiped out with filter paper, and then the tissues were submerged in the optimal cutting temperature compound (OCT; 4583; Sakura, Japan) to observe the effectiveness of infection. Allow the OCT to solidify at −20°C. After sectioning into pieces of 10 µm thickness on a rapid sectioning cryostat (CM1950; Leica, Germany), the intensity of EGFP green fluorescent signal was observed by confocal microscopy (AX NIS‐Elements 5.4; Nikon). The third part's mucosa layer was isolated for WB experiment. Tff2 protein expression was quantified in the scramble and Tff2‐KD groups to assess the efficiency of Tff2‐KD. The employed antibodies are listed in Tables S1 and S3.

### 4.10 Serum Levels of d‐Lactic Acid and Diamine Oxidase in Tff2‐KD Rats

Serum D‐lactic acid (D‐La) concentration was detected using a D‐La assay kit with WST‐8 (S0204S; Beyotime, Shanghai, China), and diamine oxidase (DAO) concentration was detected using a rat DAO ELISA kit (E‐EL‐R3013; Elabscience, Wuhan, China). The experiments were conducted step by step following the manufacturer's instructions, and the final concentrations were calculated according to the standard curve.

### 4.11 Cell Culture

GES‐1 cells were purchased from Fuheng Biology (FH0273; Shanghai, China) and cultured in Dulbecco's modified Eagle's medium (DMEM) complete medium (Fuheng Biology; FH‐GES‐1; Shanghai, China). Primary human gastric mucosal epithelial (CP‐H048) cells and primary human gastric mucosal epithelial cell complete medium (CM‐H048) were purchased from Procell (Wuhan, Hubei, China). Under all conditions, the cells were incubated at 37°C in a 5% CO_2_‐containing atmosphere.

To mimic the rat model, both cells were given 100 µmol/L nitrate pretreatment 24 h in advance of EtOH stimulation. After 7% (v/v) EtOH‐containing medium incubated for 3 h at room temperature, cells were collected for RT‐qPCR IF staining, and Western blot analysis. The cells were categorized into the following four groups according to the presence or absence of nitrate and EtOH:

1. Ctrl: complete medium + 7% (v/v) PBS incubation;
2. Ctrl + Nit: 100 µmol/L sodium nitrate treatment + 7% (v/v) PBS incubation;
3. EtOH: complete medium + 7% (v/v) EtOH incubation;
4. EtOH + Nit: 100 µmol/L sodium nitrate treatment + 7% (v/v) EtOH incubation.

Since CP‐H048 is primary cells derived from normal gastric mucosal epithelial tissue, it can only be passaged three to four times. Only the wound healing assay and RT‐qPCR were conducted to validate vital results of GES‐1 cells.

### 4.12 Wound Healing Assay

To minimize errors caused by manual scratching, a culture insert (80366; Ibidi, Germany) was utilized to conduct wound healing assay. In the nitrate pretreatment experiment, the culture conditions and grouping of GES‐1 cells and CP‐H048 cells were the same as before. The main steps are as follows:

In each well of the culture inserts, 6.5–7 × 10^4^ cells per well were planted. The nitrate groups were pretreated with 100 µmol/L nitrate medium for 24 h, and a cell‐free gap of 500 µm was created by removing the culture inserts. After two washes with PBS, the EtOH groups were treated with a 7% (v/v) EtOH‐containing medium and incubated for 3 h. Cells in the control and control + Nit groups were treated with a medium containing an equivalent volume of PBS at the same time. Finally, the 7% (v/v) EtOH‐containing medium was replaced with serum‐free medium to observe the cell migration ability.

Nitrate therapy experiments were performed with GES‐1 cells. Nitrate pretreatment prior to EtOH incubation was canceled. Following scratching and EtOH incubation, 100 µmol/L nitrate was administrated to the serum‐free medium. Based on the nitrate and EtOH treatment conditions, the cells were divided into the following four groups: control, control + Nit‐treated, EtOH, and EtOH + Nit‐treated group.

An inverted microscope (IX71; Olympus, Japan) was utilized to capture images following the generation of scratches at 0, 24, and 48 h. A commonly utilized area quantification approach was employed to assess the cell migration rate, defined as the percentage of closed area to the initial scratch area.

### 4.13 IF Staining of GES‐1 Cells

The culture medium was removed, and the GES‐1 cells were washed thrice in PBS. The cells were fixed with 4% PFA for 20 min, and incubated with 0.3% Triton X‐100 (HFH10; Thermo, USA) for 20 min. Subsequently, GES‐1 cells were blocked with 1% goat serum for 1 h. The cells were incubated with NICD or pMLC antibody at 4°C overnight after the goat serum was discarded.

The primary antibody was washed thrice with PBS, and secondary antibody was incubated. After 10‐min DAPI staining, cell slides were mounted with ProLong Gold Antifade Mountant (P36930; Thermo, USA). A Nikon confocal microscope was used for image acquisition. Antibodies used are listed in Table S1.

### 4.14 *TFF2* siRNA‐KD in GES‐1 Cells

*TFF2* siRNA and NC siRNAs were obtained from Genomeditech (Shanghai, China). The sequences are as follows:

5′‐GCUGUUUCGACUCCAGUGU(dT)(dT)‐3′,
5′‐ACACUGGAGUCGAAACAGC(dT)(dT)‐3′.

NC siRNA or *TFF2* siRNA was transfected at 50 nmol/L using LipoRNAiMAX (L3000075; Thermo, USA) per manufacturer instructions. The transfection efficiency of *TFF2* siRNA was validated using RT‐qPCR. At 48 h after transfection and nitrate treatment, the cells were treated with a 7% (v/v) EtOH culture medium.

### 4.15 Supershift EMSA

The EMSA procedure was implemented according to a previously described procedure [75]. A 5′‐biotinylated oligonucleotide (5′‐ATG CAT GCA TGC ATG CAT GCA TGC‐3′) was used as the probes. Three TFBSs of TFF2 promoter with the highest scores were simultaneously mutated in the mutant probes. The KOD‐Plus Kit (KOD‐201; Toyobo, Shanghai, China) was used to mark the wild‐type and mutated oligonucleotides. The probes were incubated with the GES‐1 cells extract and RBPJ antibody (5313; Cell Signaling Technology, USA) at room temperature for 50 min. To comprehend the specificity of the DNA/protein binding reactions, competition assays were performed with 50/100 excessive unlabeled cold probes. The entire reaction mixture was run on a nondenaturing 0.5×TBE 6% polyacrylamide gel for 1 h at 80 V at 4°C and then transferred onto a biodyne B nylon membrane (Pall Corporation, USA). The nylon membrane was incubated with 1:3000 diluted HRP‐conjugated Streptavidin (SA00001‐0; proteintech, Wuhan, China) antibody at room temperature for 20 min. Signals were visualized with warm water‐dissolved developing powder (YA0370; Solarbio, Beijing, China) and fixer (YA0380, Solarbio).

### 4.16 DLR Gene Assay

The binding sites of RBPJ on the TFF2 promoter were predicted in a human transcription factor database (http://bioinfo.life.hust.edu.cn/HumanTFDB/,Human TFDB). The firefly luciferase plasmids TFF2 (Full, Mut1, Mut2, and Mut3) promoter were constructed on pGL3‐Basic. Transcription factor RBPJ‐overexpressing plasmids were constructed on pcDNA3.1 vector. NICD‐overexpressing plasmids were constructed on GV341 vector. Renilla plasmids were cotransfected as internal reference to eliminate transfection efficiency differences. All plasmids were designed and purchased from Tsingke (Beijing, China). GES‐1 cells were cotransfected with above three plasmids using the Lipofectamine 3000 transfection reagent (L3000015; Thermo). A dual luciferase reporter assay system was applied to measure luciferase activity 48 h after transfection (E1910; Promega, WI, USA). Normalized firefly luciferase activity was compared between the two groups. Data are presented as fold change relative to the vector groups.

### 4.17 Cleavage Under Targets and Tagmentation qPCR

A protein‐DNA interaction assay, cleavage under targets and tagmentation (CUT&Tag) qPCR, was conducted with GES‐1 cells. With or without nitrate pretreatment, GES‐1 cells were collected at 100,000 cells/group. RBPJ and IgG antibodies were used according to a previously described protocol. Hyperactive Universal CUT&Tag Assay Kit for Illumina Pro (TD904; Vazyme) and CUT&Tag Stop Buffer for qPCR (TD904‐C1; Vazyme) was used to perform CUT&Tag qPCR experiments. The experiments were strictly conducted per manufacturer instructions. qPCR primers were designed by an experienced technician. The sequences are as follows:

F: CGATGCCACTCTATTGCAGA, R: TCTGCTGACTGTTCACCACT.

### 4.18 Notch‐Activated Wound Healing Assay

GES‐1 cells were seeded into six cells plate at 2 × 10^5^ cells/well and treated with 10 µmol/L N2‐(4‐isopropylphenyl)‐5‐(3‐methoxyphenoxy) quinazoline‐2,4‐diamine (code‐named Yhhu‐3792, HY‐120782; MCE, Shanghai, China) for 2 days. A wound migration model of cultured cells was used, as described above, to explore the effect of Notch pathway activation on GES‐1 cell migration. RT‐qPCR were conducted to observe the activation efficiency. The primer sequences for Notch pathway genes and *TFF2* are listed in Table S2.

### 4.19 NICD Deprivation of GES‐1 Cells

(3,5‐Difluorophenacetyl)‐l‐alanyl‐S‐phenylglycine‐2‐butyl ester (code‐named DAPT, HY‐13027; MCE) is an effective and orally active γ‐secretase inhibitor, which is widely used in the inhibition and deprivation experiments of NICD. To assess the function of NICD on RBPJ's inhibition of TFF2 expression, 2 × 10^5^ cells per well were plated in 12‐well plates and treated either with 2 µmol/L DMSO vehicle control or 2 µmol/L DAPT. Meanwhile, RBPJ overexpressing plasmid was transfected with the same method using the Lipofectamine 3000 transfection reagent. After 2 days of cotreatment, GES‐1 cells were lysed. Protein and RNA were extracted using the same method described above.

### 4.20 in situ PLA

The in situ PLA was performed on fixed GES‐1 cells following the manufacturer's protocol (DUO82049, DUO92008; Merck). Following incubation with primary NICD (NBP1‐48289; Novus Biologicals, USA) and RBPJ (720219; Thermo, USA) antibodies, a pair of Duolink PLA probes—anti‐mouse MINUS (DUO92004; Merck) and anti‐rabbit PLUS (DUO92002; Merck)—were applied for 60 min. Ligase was added for 30 min followed by amplification of signal for 100 min using polymerase. Coverslips were then mounted using the Duolink in situ mounting medium with DAPI (DUO82040; Merck) and sealed with clear nail polish. Fluorescent images were captured using a Nikon Confocal Laser Scanning Microscope with the NIS‐Elements Viewer software.

### 4.21 Statistical Analysis

Blind data collection and analysis were performed. For the significance analysis of multiple comparisons, a one‐way analysis of variance with a posthoc test was used for normal distribution and the Kruskal–Wallis test for others. All data are presented as the mean ± SD and processed with SPSS 23.0, and GraphPad Prism 9.5. *p < 0.05, **p < 0.01, and ***p < 0.001 were considered statistically significant.

## Author Contributions

Ying Liu and Xin Wen performed the experiments, analyzed the data, and wrote the manuscript. Yuxuan Lin, Chunmei Zhang, and Jinsong Wang prepared the reagents used for the experiments and set up the experimental systems. Renhong Yan and Mo Chen administered EMSA. Guangyong Sun, Dong Zhang, Songlin Wang, and Shaorong Li led the project, interpreted the data, and revised the manuscript. All authors have read and approved the final manuscript.

## Funding

This work was supported by the National Natural Science Foundation of China (82201054, 82030031, L2224038), the Beijing Municipal Government grant (Beijing Laboratory of Oral Health, PXM2021‐014226‐000041), the Beijing Municipal Education Commission (119207020201), the Beijing Stomatological Hospital, Capital Medical University Young Scientist Program (YSP202102), the Innovation Research Team Project of Beijing Stomatological Hospital, Capital Medical University (CXTD202201), the Chinese Research Unit of Tooth Development and Regeneration, Academy of Medical Sciences (2019‐12M‐5‐031), the Beijing Municipal Government (Beijing Scholar Program, PXM2020_014226_000005 and PXM2021_014226_000020), the Beijing Municipal Colleges and Universities High Level Talents Introduction and Cultivate Project‐Beijing Great Wall Scholar Program (CIT&TCD 20180332), State Key Laboratory of Oral Diseases, Sichuan University (SKLOD2023OF13), the National Key Research and development Program (2022YFA1104401), and Young Elite Scientists Sponsorship Program of the Beijing High Innovation Plan. Renhong Yan is an investigator of SUSTech Institute for Biological Electron Microscopy.

## Ethics Statement

All procedures were sanctioned by the Institutional Animal Care and Use Committee of Capital Medical University (Ethical Approval: AEEI‐2023‐110). The study was performed in accordance with the institutional guidelines for the care and use of laboratory animals. All procedures complied with the ARRIVE guidelines and were conducted to minimize animal suffering.

## Conflicts of Interest

The authors declare no conflicts of interest.

## Supporting information




**Figure S1**: Scramble and Tff2 knockdown rats were established by AAV infection. (A) Representative cryosection images of scramble and Tff2‐KD rats’ gastric tissue. Scale bar = 100 µm. (B) Analysis of EGFP fluorescence intensity in gastric tissue between scramble and Tff2‐KD rats. (C and D) Representative immunoblotting band and gray value analyses of gastric mucosa Tff2 expression in scramble and Tff2‐KD rats. Quantitative data are expressed as the mean ± SD. **p < 0.01, and ns denotes no significance. Tff2; trefoil peptide factor 2; AAV, adeno‐associated virus; KD, knockdown; EGFP, enhanced green fluorescent protein; MFI, mean fluorescence intensity; SD, standard deviation.
**Figure S2**: Tff2 knockdown eliminates nitrate's function of anti‐inflammatory and epithelial barrier maintenance. (A) IF staining of TNF‐α (green) and DAPI (blue). (B) IF staining of IL‐1β (red) and DAPI (blue). (C) IF staining of occludin (green) and DAPI (blue). (D) IF staining of ZO‐1 (red) and DAPI (blue). (E–H) IF analysis of TNF‐α, IL‐1β, occludin, ZO‐1 with MFI. Scale bar = 200 µm. (I) The d‐La levels of the serum in Tff2‐KD and scramble groups with ethanol gavage. (J) The DAO levels of the serum in Tff2‐KD and scramble groups with ethanol gavage. Quantitative data are expressed as the mean ± SD. ***p < 0.001, and ns denotes no significance. Tff2, trefoil peptide factor 2; IF, immunofluorescence; EtOH, ethanol; Nit, nitrate; TNF‐α, tumor necrosis factor alpha; IL‐1β, interleukin‐1β; ZO‐1, zonula occludens‐1; DAPI, 2‐(4‐amidinophenyl)‐6‐indolecarbamidine dihydrochloride; MFI, mean fluorescence intensity; d‐La, d‐lactic acid; DAO, diamine oxidase; KD, knockdown; SD, standard deviation.
**Figure S3**: Both post‐ and pretreatment of nitrate exert a similar promotion effect on migration. (A–C) Postnitrate treatment promotes GES‐1 cells’ migration (A) Images of the postnitrate treatment scratch healing process of GES‐1 cells. Scale bar = 400 µm. (B) Quantitative analysis of the migration rate at 24 h in (A). (C) Quantitative analysis of the migration rate at 48 h in (A). (D–I) Prenitrate treatment promotes the migration of CP‐H048 cells and regulates the expression of migration‐related genes. (D) Images of the scratch healing process of CP‐H048 cells with prenitrate treatment. Scale bar = 400 µm. (E) Quantitative analysis of the migration rate at 24 h in (D). (F) Quantitative analysis of the migration rate at 48 h in (D). (G–I) RT‐qPCR analysis of *NOTCH1*, *RBPJ*, and *TFF2* mRNA of CP‐H048 cells. Target gene expression was normalized to *GAPDH* mRNA and expressed as fold change relative to the Ctrl group. Quantitative data are expressed as the mean ± SD. *p < 0.05, **p < 0.01, ***p < 0.001. GES‐1, human gastric epithelial; EtOH, ethanol; Nit, nitrate; RBPJ, recombination signal binding protein for immunoglobulin kappa J region; TFF2, trefoil peptide factor 2; SD, standard deviation.
**Figure S4**: Analysis of transcription factors with binding potential to the TFF2 promoter by RT‐qPCR in GES‐1 cells. (A) RT‐qPCR analysis of *TFF2* mRNA of vector and *TFF2* knockdown GES‐1 cells. Target gene expression was normalized to *GAPDH* mRNA and expressed as fold change relative to the negative control group. (B–P) RT‐qPCR analysis of predicted TFs mRNA of GES‐1 cells and EtOH‐treated GES‐1 cells with/without nitrate treatment. Target gene expression was normalized to *GAPDH* mRNA and expressed as fold change relative to the Ctrl group. Quantitative data are expressed as the mean ± SD. ***p < 0.001, **p < 0.01, ns denotes no significance. TFF2, trefoil peptide factor 2; SiRNA, small interfering RNA; GES‐1, human gastric epithelial; EtOH, ethanol; Nit, nitrate; Ctrl, control; RT‐qPCR, real‐time quantitative polymerase chain reaction; TFs, transcription factors; FOS, Fos proto‐oncogene; JUN, Jun proto‐oncogene; MYC, MYC proto‐oncogene; ERG, ETS transcription factor ERG; CEBPB, CCAAT enhancer binding protein beta; CEBPD, CCAAT enhancer binding protein delta; PAX2, PAIRED BOX 2; POU2F2, POU class 2 homeobox 2; POU2F3, POU class 2 homeobox 3; KLF4, Kruppel like factor 4; KLF9, Kruppel like factor 9; TCF7L1, transcription factor 7 like 1; TCF7L2, transcription factor 7 like 2; GATA4, GATA binding protein 4; RBPJ, recombination signal binding protein for immunoglobulin kappa J region.
**Figure S5**: Schematic representation of the wild‐type and mutant RBPJ‐binding site promoter constructs for the dual‐luciferase reporter assay. (A) Design of the mutations within the TFF2 promoter region of luciferase constructs in firefly luciferase plasmids. The transcription start site (ATG) is indicated. Top three potential RBPJ binding sites (Site 1: −1824/−1819; Site 2: −1643/−1638; Site 3: −1599/−1594) were identified. The core sequences of the wild‐type (WT) sites and their mutant (Mut) counterparts are shown below. Mutated core sequences within each site are highlighted in red. The promoter fragments (“Full” and mutants “Mut1”, “Mut2”, “Mut3”) were cloned into a luciferase reporter vector to assess the transcriptional activity driven by different role of each site. Mut, mutant; TFF2, trefoil peptide factor 2; GES‐1, human gastric epithelial; RBPJ, recombination signal binding protein for immunoglobulin kappa J region; DLR, dual‐luciferase reporter.
**Figure S6**: Regulatory efficiency of reagents targeting the Notch signaling pathway. (A–C) RT‐qPCR analysis of mRNAs in Notch pathway between untreated and Yhhu‐3792‐activated GES‐1 cells. (D) RT‐qPCR analysis of *TFF2* mRNA of untreated and Yhhu‐3792‐activated GES‐1 cells. (E) Images of the scratch healing process of Yhhu‐3792‐activated GES‐1 cells in Ibidi culture inserts. Scale bar = 500 µm. (F and G) Quantitative analysis of the migration rate at 24 and 48 h in (I). (H and I) Representative immunoblotting band of NICD protein and gray value analyses of DMSO or DAPT‐treated GES‐1 cells. (J) RT‐qPCR analysis of *RBPJ* mRNA in GES‐1 cells transfected with an RBPJ overexpression plasmid (RBPJ OE) or an empty vector control (vector). (K and L) Representative immunoblotting band of NICD protein and gray value analyses in GES‐1 cells transfected with an NICD overexpression plasmid (NICD OE) or an empty vector control (vector). Target gene expression was normalized to *GAPDH* mRNA and expressed as fold change relative to the blank GES‐1 cells. Quantitative data are expressed as the mean ± SD. **p < 0.01, ***p < 0.001, and ns denotes no significance. TFF2, trefoil peptide factor 2; EtOH, ethanol; Nit, nitrate; Ctrl, control; KD, knockdown; OE, over expression; GES‐1, human gastric epithelial; RT‐qPCR, real‐time quantitative polymerase chain reaction; RBPJ, recombination signal binding protein for immunoglobulin kappa J region; MAML1, mastermind like transcriptional coactivator 1; NICD, Notch intracellular structural domain; Nit, nitrate; Yhhu‐3792, N2‐(4‐isopropylphenyl)‐5‐(3‐methoxyphenoxy) quinazoline‐2,4‐diamine; DAPT, (3,5‐difluorophenacetyl)‐l‐alanyl‐S‐phenylglycine‐2‐butyl ester.
**Table S1**: Immunofluorescence staining antibodies.
**Table S2**: RT‐qPCR primer sequences.
**Table S3**: Western blot primary antibodies.

## Data Availability

Data in support of the findings of this study are available from the corresponding author upon reasonable request. The RNA‐seq datasets supporting this study are available in the China National GeneBank Sequence Archive (CNSA) under accession number CNP0007543.
