# Supplementary material for: Nitrate Enhances Gastric Mucosa Defense and Repair Process in Ethanol‐Induced Gastric Ulcer Rats via the Notch–Tff2 Pathway
Source: MedComm (2020). 2026 Feb 5;7(2):e70628. doi: 10.1002/mco2.70628 (PMC12877318; doi:10.1002/mco2.70628)
Supplement: Supplementary file 1 — Figure S1: Scramble and Tff2 knockdown rats were established by AAV infection. (A) Representative cryosection images of scramble and Tff2‐KD rats’ gastric tissue. Scale bar = 100 µm. (B) Analysis of EGFP fluorescence intensity in gastric tissue between scramble and Tff2‐KD rats. (C and D) Representative immunoblotting band and gray value analyses of gastric mucosa Tff2 expression in scramble and Tff2‐KD rats. Quantitative data are expressed as the mean ± SD. **p < 0.01, and ns denotes no significance. Tff2; trefoil peptide factor 2; AAV, adeno‐associated virus; KD, knockdown; EGFP, enhanced green fluorescent protein; MFI, mean fluorescence intensity; SD, standard deviation. Figure S2: Tff2 knockdown eliminates nitrate's function of anti‐inflammatory and epithelial barrier maintenance. (A) IF staining of TNF‐α (green) and DAPI (blue). (B) IF staining of IL‐1β (red) and DAPI (blue). (C) IF staining of occludin (green) and DAPI (blue). (D) IF staining of ZO‐1 (red) and DAPI (blue). (E–H) IF analysis of TNF‐α, IL‐1β, occludin, ZO‐1 with MFI. Scale bar = 200 µm. (I) The d‐La levels of the serum in Tff2‐KD and scramble groups with ethanol gavage. (J) The DAO levels of the serum in Tff2‐KD and scramble groups with ethanol gavage. Quantitative data are expressed as the mean ± SD. ***p < 0.001, and ns denotes no significance. Tff2, trefoil peptide factor 2; IF, immunofluorescence; EtOH, ethanol; Nit, nitrate; TNF‐α, tumor necrosis factor alpha; IL‐1β, interleukin‐1β; ZO‐1, zonula occludens‐1; DAPI, 2‐(4‐amidinophenyl)‐6‐indolecarbamidine dihydrochloride; MFI, mean fluorescence intensity; d‐La, d‐lactic acid; DAO, diamine oxidase; KD, knockdown; SD, standard deviation. Figure S3: Both post‐ and pretreatment of nitrate exert a similar promotion effect on migration. (A–C) Postnitrate treatment promotes GES‐1 cells’ migration (A) Images of the postnitrate treatment scratch healing process of GES‐1 cells. Scale bar = 400 µm. (B) Quantitative analysis of the migration rate at 24 h [file MCO2-7-e70628-s001.docx]

**Nitrate enhances gastric mucosa defence and repair process in ethanol-induced gastric ulcer rats via the Notch-Tff2 pathway**

Ying Liu^1,2#^, Xin Wen ^1,2#^, Yuxuan Lin^1,2^, Chunmei Zhang^1,2^, Jinsong Wang^1, 3^, Guangyong Sun^1,2,4^, Dong Zhang^1,2,4^, Renhong Yan^5,6^, Mo Chen^7^, Songlin Wang^1,2,3,8,9*^ and Shaorong Li^1,2,10*^

^1^ Salivary Gland Disease Center and Beijing Key Laboratory of Tooth Regeneration and Function Reconstruction, Beijing Laboratory of Oral Health and School of Stomatology, Capital Medical University, Beijing, China.

^2^ Immunology Research Center for Oral and Systemic Health, Beijing Friendship Hospital, Capital Medical University, Beijing, China.

^3^ Department of Biochemistry and Molecular Biology, School of Basic Medical Sciences, Capital Medical University, Beijing, China.

^4^ Medical Research Center, Beijing Institute of Respiratory Medicine and Beijing Chao-Yang Hospital, Capital Medical University, Beijing, China.

^5^ Department of Biochemistry, School of Medicine, Southern University of Science and Technology, Shenzhen, Guangdong Province, China.

^6^ Key University Laboratory of Metabolism and Health of Guangdong, SUSTech Homeostatic Medicine Institute, Institute for Biological Electron Microscopy, Southern University of Science and Technology, Shenzhen, Guangdong Province, China.

^7^ Department of Pharmacology, Joint Laboratory of Guangdong-Hong Kong Universities for Vascular Homeostasis and Diseases, School of Medicine and SUSTech Homeostatic Medicine Institute (SHMI), Southern University of Science and Technology, Shenzhen, China.

^8^ Laboratory for Oral and General Health Integration and Translation, Beijing Tiantan Hospital, Capital Medical University, Beijing, China.

^9^ Laboratory of Homeostatic Medicine, School of Medicine, Southern University of Science and Technology, Shenzhen, China.

^10^ Department of Endodontics, School of Stomatology, Capital Medical University, Beijing, China.

# Ying Liu and Xin Wen contributed equally to this work.

*Correspondence

Songlin Wang, Salivary Gland Disease Center and Beijing Key Laboratory of Tooth Regeneration and Function Reconstruction, Beijing Laboratory of Oral Health and School of Stomatology, Immunology Research Center for Oral and Systemic Health, Beijing Friendship Hospital, Department of Biochemistry and Molecular Biology, School of Basic Medical Sciences, Laboratory for Oral and General Health Integration and Translation, Beijing Tiantan Hospital, Capital Medical University, Beijing, China.

Email: [slwang@ccmu.edu.cn](mailto:slwang@ccmu.edu.cn)

Shaorong Li, Salivary Gland Disease Center and Beijing Key Laboratory of Tooth Regeneration and Function Reconstruction, Beijing Laboratory of Oral Health and School of Stomatology, Immunology Research Center for Oral and Systemic Health, Beijing Friendship Hospital, Department of Endodontics, School of Stomatology, Capital Medical University, Beijing, China.

Email: [lishaorong@ccmu.edu.cn](mailto:lishaorong@ccmu.edu.cn)

**Supplementary Figures**


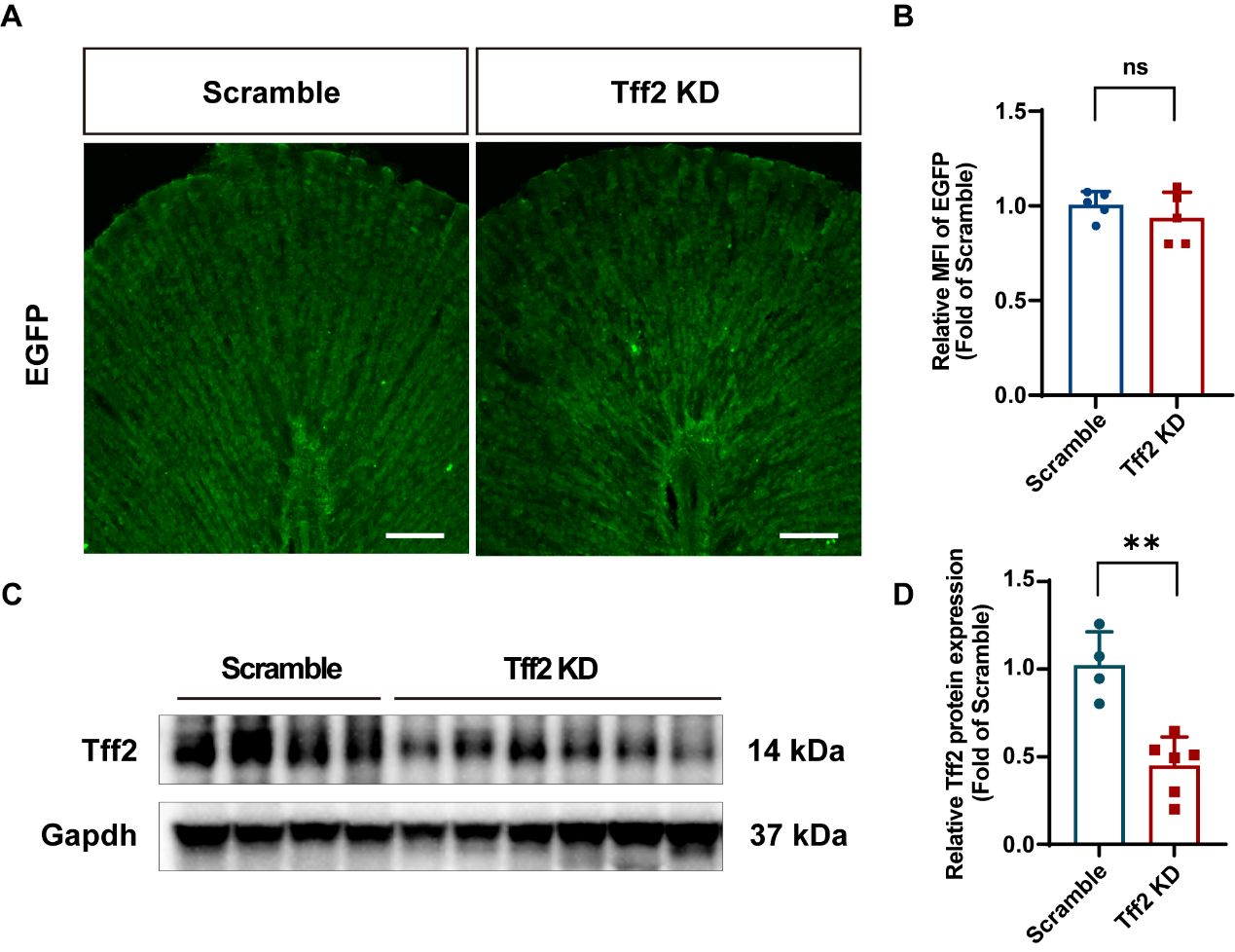


**Figure S1|** Scramble and Tff2 knockdown rats were established by AAV infection. **(A)** Representative cryosection images of Scramble and Tff2 KD rats’ gastric tissue. Scale bar = 100 μm. **(B)** Analysis of EGFP fluorescence intensity in gastric tissue between Scramble and Tff2 KD rats. **(C-D)** Representative immunoblotting band and gray value analyses of gastric mucosa Tff2 expression in Scramble and Tff2 KD rats. Quantitative data are expressed as the mean ± SD. ***P*＜0.01, and ns denotes no significance.

Tff2; trefoil peptide factor 2; AAV, adeno-associated virus; KD, knockdown; EGFP, Enhanced Green Fluorescent Protein; MFI, mean fluorescence intensity; SD, standard deviation.


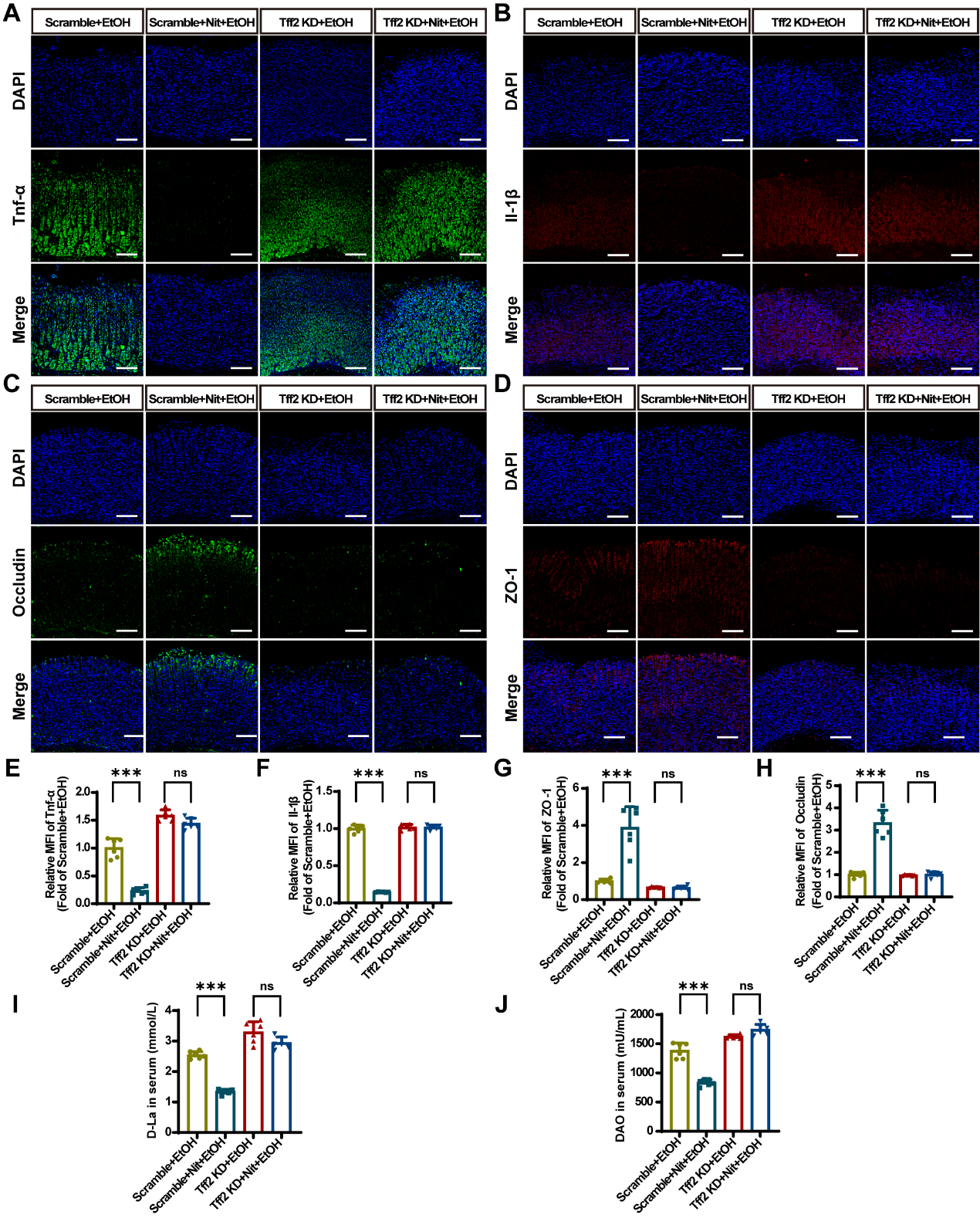
**Figure S2|** Tff2 knockdown eliminates nitrate's function of anti-inflammatory and epithelial barrier maintenance. **(A)** IF staining of TNF-α (green) and DAPI (blue). **(B)** IF staining of IL-1β (red) and DAPI (blue). **(C)** IF staining of Occludin (green) and DAPI (blue). **(D)** IF staining of ZO-1 (red) and DAPI (blue). **(E-H)** IF analysis of TNF-α, IL-1β, Occludin, ZO-1 with MFI. Scale bar = 200 μm. **(I)** The D-La levels of the serum in Tff2 KD and Scramble groups with ethanol gavage. **(J)** The DAO levels of the serum in Tff2 KD and Scramble groups with ethanol gavage. Quantitative data are expressed as the mean ± SD. ****P*＜0.001, and ns denotes no significance.

Tff2, trefoil peptide factor 2; IF, immunofluorescence; EtOH, ethanol; Nit, nitrate; TNF-α, tumor necrosis factor alpha; IL-1β, interleukin-1β; ZO-1, zonula occludens-1; DAPI, 2-(4-Amidinophenyl)-6-indolecarbamidine dihydrochloride; MFI, mean fluorescence intensity; D-La, D-Lactic acid; DAO, diamine oxidase; KD, knockdown; SD, standard deviation.


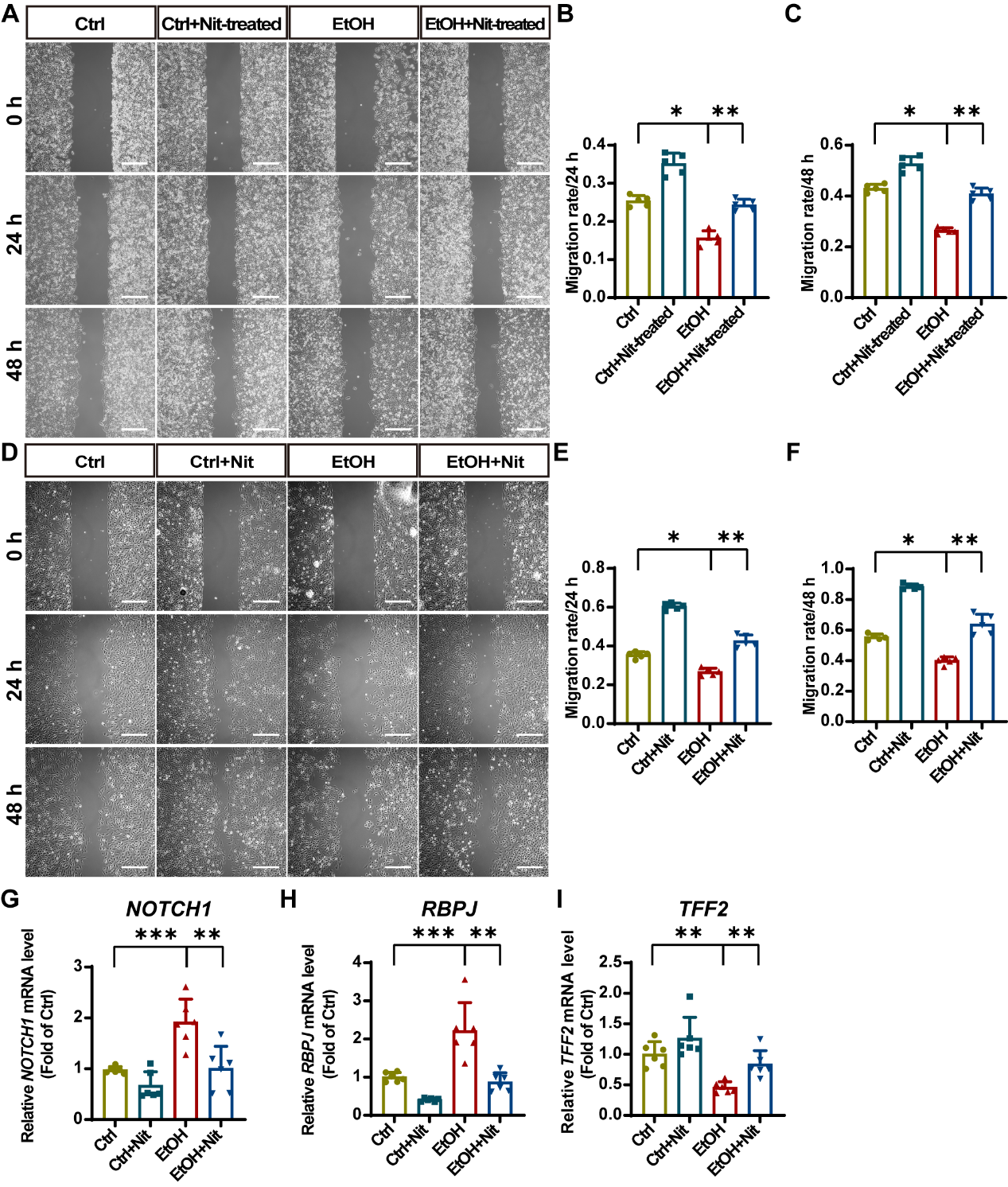


**Figure S3|** Both post- and pre-treatment of nitrate exert a similar promotion effect on migration. **(A-C)** Post-nitrate treatment promotes GES-1 cells’ migration **(A)** Images of the post-nitrate treatment scratch healing process of GES-1 cells. Scale bar = 400 μm. **(B)** Quantitative analysis of the migration rate at 24 h in (A). **(C)** Quantitative analysis of the migration rate at 48 h in (A). **(D-I)** Pre-nitrate treatment promotes the migration of CP-H048 cells and regulates the expression of migration-related genes. **(D)** Images of the scratch healing process of CP-H048 cells with pre-nitrate treatment. Scale bar = 400 μm. **(E)** Quantitative analysis of the migration rate at 24 h in (D). **(F)** Quantitative analysis of the migration rate at 48 h in (D). **(G-I)** RT-qPCR analysis of *NOTCH1, RBPJ* and *TFF2* mRNA of CP-H048 cells. Target gene expression was normalized to *GAPDH* mRNA and expressed as fold change relative to the Ctrl group. Quantitative data are expressed as the mean ± SD. **P*＜0.05, ***P*＜0.01, ****P*＜0.001.

GES-1, human gastric epithelial; EtOH, ethanol; Nit, nitrate; RBPJ, recombination signal binding protein for immunoglobulin kappa J region; TFF2, trefoil peptide factor 2; SD, standard deviation.


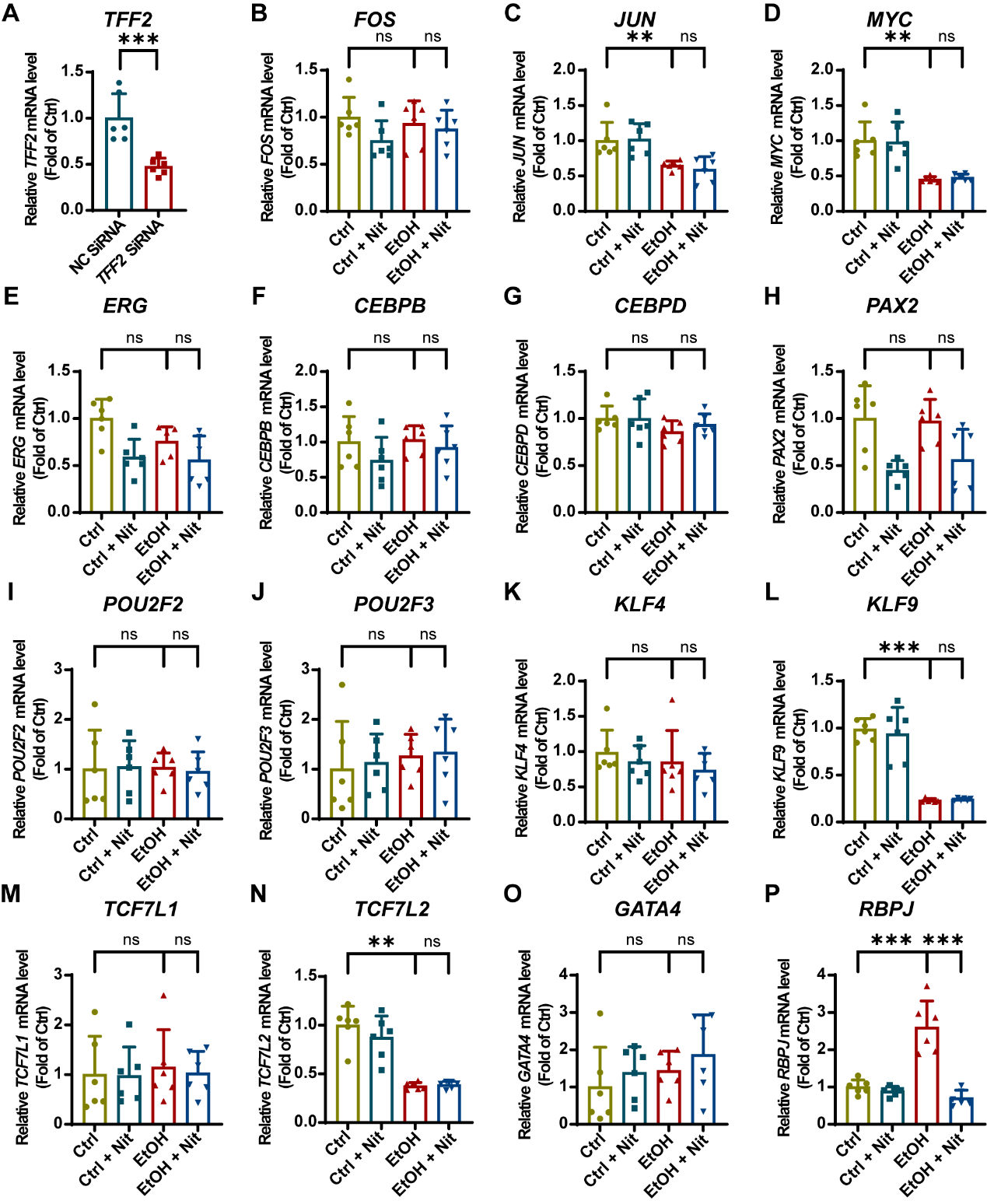


**Figure S4|** Analysis of transcription factors with binding potential to the TFF2 promoter by RT-qPCR in GES-1 cells. **(A)** RT-qPCR analysis of *TFF2* mRNA of vector and *TFF2* knockdown GES-1 cells. Target gene expression was normalized to *GAPDH* mRNA and expressed as fold change relative to the Negative Control group. **(B-P)** RT-qPCR analysis of predicted TFs mRNA of GES-1 cells and EtOH-treated GES-1 cells with/without nitrate treatment. Target gene expression was normalized to *GAPDH* mRNA and expressed as fold change relative to the Ctrl group. Quantitative data are expressed as the mean ± SD. ****P*＜0.001, ***P*＜0.01, ns denotes no significance.

TFF2, trefoil peptide factor 2; SiRNA, small interfering RNA ; GES-1, human gastric epithelial; EtOH, ethanol; Nit, nitrate; Ctrl, control; RT-qPCR, real-time quantitative polymerase chain reaction; TFs, transcription factors; FOS, Fos proto-oncogene; JUN, Jun proto-oncogene; MYC, MYC proto-oncogene; ERG, ETS transcription factor ERG; CEBPB, CCAAT Enhancer Binding Protein Beta; CEBPD, CCAAT Enhancer Binding Protein Delta; PAX2, Paired Box 2; POU2F2, POU Class 2 Homeobox 2; POU2F3, POU Class 2 Homeobox 3; KLF4, Kruppel Like Factor 4; KLF9, Kruppel Like Factor 9; TCF7L1, Transcription Factor 7 Like 1; TCF7L2, Transcription Factor 7 Like 2; GATA4, GATA Binding Protein 4; RBPJ, recombination signal binding protein for immunoglobulin kappa J region.


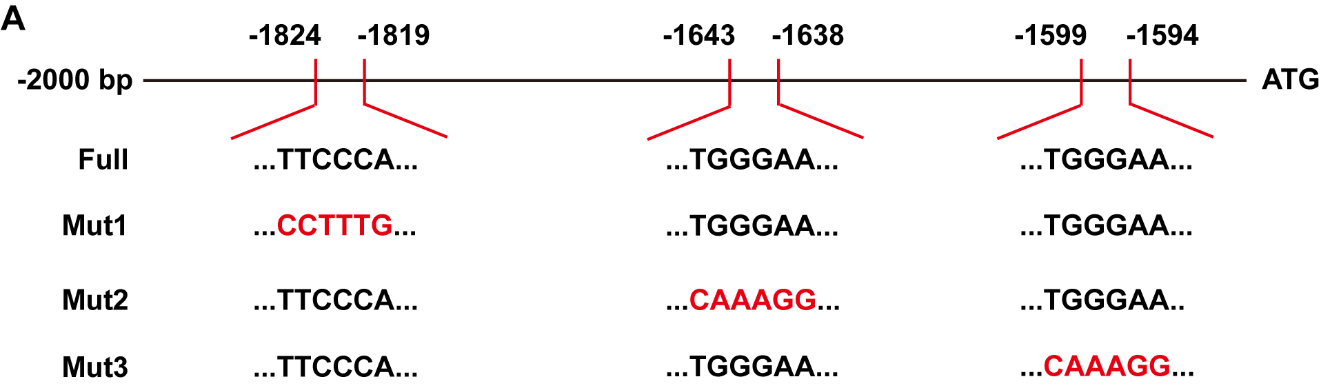


**Figure S5|** Schematic representation of the wild-type and mutant RBPJ-binding site promoter constructs for the dual-luciferase reporter assay. **(A)** Design of the mutations within the TFF2 promoter region of luciferase constructs in firefly luciferase plasmids. The transcription start site (ATG) is indicated. Top three potential RBPJ binding sites (Site 1: -1824/-1819; Site 2: -1643/-1638; Site 3: -1599/-1594) were identified. The core sequences of the wild-type (WT) sites and their mutant (Mut) counterparts are shown below. Mutated core sequences within each site are highlighted in red. The promoter fragments ("Full" and mutants "Mut1", "Mut2", "Mut3") were cloned into a luciferase reporter vector to assess the transcriptional activity driven by different role of each site. Mut, mutant; TFF2, trefoil peptide factor 2; GES-1, human gastric epithelial; RBPJ, recombination signal binding protein for immunoglobulin kappa J region; DLR, dual-luciferase reporter.


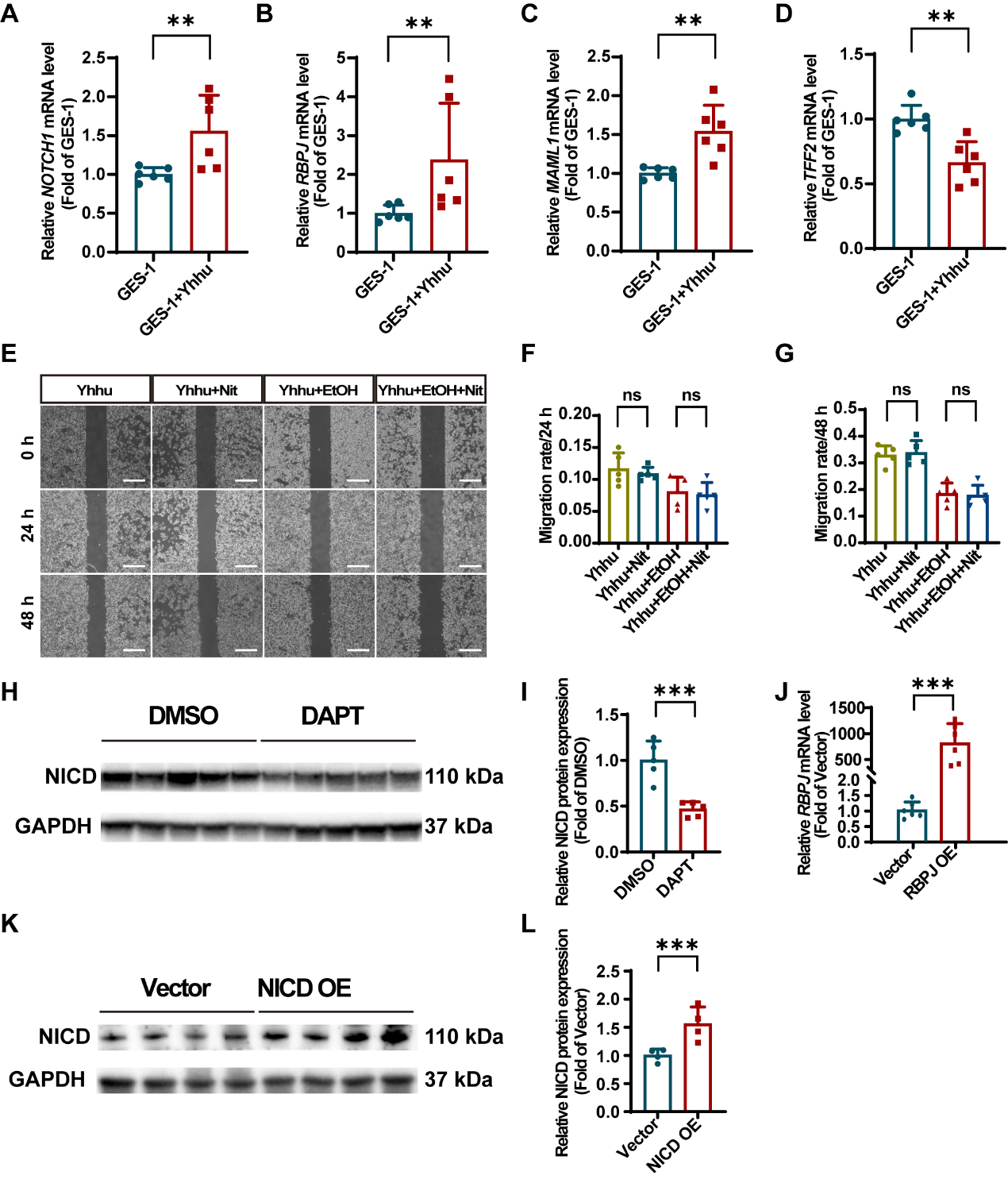


**Figure S6|** Regulatory efficiency of reagents targeting the Notch signaling pathway. **(A-C)** RT-qPCR analysis of mRNAs in Notch pathway between untreated and Yhhu-3792-activated GES-1 cells. **(D)** RT-qPCR analysis of *TFF2* mRNA of untreated and Yhhu-3792-activated GES-1 cells. **(E)** Images of the scratch healing process of Yhhu-3792-activated GES-1 cells in Ibidi culture inserts. Scale bar = 500 μm. **(F-G)** Quantitative analysis of the migration rate at 24 and 48 h in (I). **(H-I)** Representative immunoblotting band of NICD protein and gray value analyses of DMSO or DAPT-treated GES-1 cells. **(J)** RT-qPCR analysis of *RBPJ* mRNA in GES-1 cells transfected with an RBPJ overexpression plasmid (RBPJ OE) or an empty vector control (Vector). **(K-L)** Representative immunoblotting band of NICD protein and gray value analyses in GES-1 cells transfected with an NICD overexpression plasmid (NICD OE) or an empty vector control (Vector). Target gene expression was normalized to *GAPDH* mRNA and expressed as fold change relative to the blank GES-1 cells. Quantitative data are expressed as the mean ± SD. ***P*＜0.01, ****P*＜0.001, and ns denotes no significance.

TFF2, trefoil peptide factor 2; EtOH, ethanol; Nit, nitrate; Ctrl, control; KD, knockdown; OE, over expression; GES-1, human gastric epithelial; RT-qPCR, real-time quantitative polymerase chain reaction; RBPJ, recombination signal binding protein for immunoglobulin kappa J region; MAML1, Mastermind Like Transcriptional Coactivator 1; NICD, Notch intracellular structural domain; Nit, nitrate; Yhhu-3792, N2-(4-isopropylphenyl)-5-(3-methoxyphenoxy) quinazoline-2,4-diamine; DAPT, (3,5-Difluorophenacetyl)-L-alanyl-S-phenylglycine-2-butyl Ester.

**Supplementary Tables**

Table S1. Immunofluorescence staining antibodies

| Protein | Antibody names | Brands | Catalog Number |
| --- | --- | --- | --- |
| CD31 | CD31 Polyclonal antibody | proteintech | 28083-1-AP |
| TGF-β1 | TGF Beta 1 Polyclonal antibody | proteintech | 21898-1-AP |
| VEGF-a | VEGFA Polyclonal antibody | proteintech | 19003-1-AP |
| TNF-α | TNF Alpha Monoclonal antibody | proteintech | 60291-1-Ig |
| IL-1β | Recombinant Anti-IL-1 beta antibody | abcam | ab283818 |
| Tff2 | Anti-TFF2 antibody [1-2#] | abcam | ab239488 |
| ZO-1 | ZO-1 Polyclonal antibody | proteintech | 21773-1-AP |
| Occludin | Occludin Monoclonal antibody | proteintech | 66378-1-Ig |
| pMLC | Phospho-Myosin Light Chain 2 (Ser19) Antibody | CST | 3671S |
| NICD | Notch-1 Antibody (OTI3E12) | NOVUS | NBP1-48289 |

Table S2. RT-qPCR primer sequences

| Gene name | Forward | Reverse |
| --- | --- | --- |
| *Ang1* | TTTGTGCTGGGTCTGGTTTCG | GCCTCTTCTCCTCATCATACTTTCG |
| *Et1* | TCTACTTCTGCCACCTGGACATC | GCATCTGTTCCCTTGGTCTGTG |
| *Il1b* | CTCACAGCAGCATCTCGACAAG | TCCACGGGCAAGACATAGGTAG |
| *Tff2* | GTGCCCCTCTCTTGGTAGTG | GACGCTTGGTTTGGAAGTG |
| *Tgfb1* | AGCAACAATTCCTGGCGTTACC | GTATTCCGTCTCCTTGGTTCAGC |
| *Tnfa* | AGATGTGGAACTGGCAGAGGAG | TCAGTAGACAGAAGAGCGTGGTG |
| *Vegfa* | CCTGGTGGACATCTTCCAGGAGTACC | GAAGCTCATCTCTCCTATGTGCTGGC |
| *Gapdh* | GAAGGGCTCATGACCACAGT | GGATGCAGGGATGATGTTCT |
| *TFF2* | ACCGAAGAAACTGTGGCTACCC | GCACCAGGGCACTTCAAAGATG |
| *NOTCH1* | TCCACCAGTTTGAATGGTCAAT | CGCAGAGGGTTGTATTGGTTC |
| *RBPJ* | AGCAATCCTTCGAGCCAATTCAAG | TGTGCTGGCGTTTGTGTAACTTC |
| *MAML1* | AATGGCGATCAACAGAATGGCTAC | GGCGTCGGCTCCAGAAGG |
| *FOS* | GTCTTCCTTCGTCTTCACCTACCC | CATTGCTGCTGCTGCCCTTG |
| *JUN* | CCAAGAACTCGGACCTCCTCAC | TGTGCCCGTTGCTGGACTG |
| *MYC* | CGTCCTCGGATTCTCTGCTCTC | TCCTCATCTTCTTGTTCCTCCTCAG |
| *ERG* | GTGGGCGGTGAAAGAATATGGC | AGAGAAGGATGTCGGCGTTGTAG |
| *CEBPB* | CTACGAGGCGGACTGCTTGG | GGTACGGGCTGAAGTCGATGG |
| *CEBPD* | CGCCATGTACGACGACGAGAG | CCCGCCTTGTGATTGCTGTTG |
| *PAX2* | AAGTGGTGGACAAGATTGCTGAATAC | CGGATGATTCTGTTGATGGAAGAGAC |
| *POU2F2* | GGTGACCTCGCCGCTCTTC | CAGGAGACTTGCTGGAGATGGAG |
| *POU2F3* | ACCAACATCCGCCTGACTCTG | ACCCTCACCACCTCCTTCTCC |
| *KLF4* | AGAGACCGAGGAGTTCAACGATC | GACGACGAAGAGGAGGCTGAC |
| *KLF9* | CCATTACAGAGTGCATACAGGTGAAC | ACACAGCGGACAGCGGAAC |
| *TCF7L1* | GGAGCCGAGCAGCGATAGC | TCTGGTTCTCCGACTCGTTGAC |
| *TCF7L2* | CACTATCGCCTGGCACCGTAG | GAACCTGGACATGGAAGCATTGAC |
| *GATA4* | CGAGATGGGACGGGTCACTATC | GGCAGTTGGCACAGGAGAGG |
| *GAPDH* | GGAGCGAGATCCCTCCAAAAT | GGCTGTTGTCATACTTCTCATGG |

Table S3. Western blot primary antibodies

| Protein | Antibody names | Brands | Catalog Number |
| --- | --- | --- | --- |
| Tff2 | Anti-TFF2 antibody [1-2#] | abcam | ab239488 |
| MLC/Mlc | Myosin Light Chain 2 (D18E2) Rabbit mAb | CST | 8505S |
| pMLC/pMlc | Phospho-Myosin Light Chain 2 (Ser19) Antibody | CST | 3671S |
| TFF2 | Recombinant Anti-TFF2 antibody [EPR23231-208] | abcam | ab267474 |
| NOTCH1/Notch1 | Notch1 (D1E11) XP® Rabbit mAb | CST | 3608S |
| NICD/Nicd | Notch-1 Antibody (OTI3E12) | abcam | Ab52301 |
| RBPJ/Rbpj | RBPSUH (D10A4) XP® Rabbit mAb | CST | 5313S |
| GAPDH/Gapdh | GAPDH Monoclonal antibody | proteintech | 60004-1-Ig |
